# Dynamic Intracellular Metabolic Cell Signaling Profiles During Ag-Dependent B-Cell Differentiation

**DOI:** 10.3389/fimmu.2021.637832

**Published:** 2021-03-30

**Authors:** Paula Díez, Martín Pérez-Andrés, Martin Bøgsted, Mikel Azkargorta, Rodrigo García-Valiente, Rosa M. Dégano, Elena Blanco, Sheila Mateos-Gomez, Paloma Bárcena, Santiago Santa Cruz, Rafael Góngora, Félix Elortza, Alicia Landeira-Viñuela, Pablo Juanes-Velasco, Victor Segura, Raúl Manzano-Román, Julia Almeida, Karen Dybkaer, Alberto Orfao, Manuel Fuentes

**Affiliations:** ^1^ Department of Medicine and Cytometry General Service-Nucleus, CIBERONC, Cancer Research Centre (IBMCC/CSIC/USAL/IBSAL), Salamanca, Spain; ^2^ Proteomics Unit, Cancer Research Centre (IBMCC/CSIC/USAL/IBSAL), Salamanca, Spain; ^3^ Department of Haematology, Aalborg University Hospital, Aalborg, Denmark; ^4^ Proteomics Platform, CIC bioGUNE, CIBERehd, ProteoRed-ISCIII, Derio, Spain; ^5^ Service of Otolaryngology and Cervical Facial Pathology, University Hospital of Salamanca, Salamanca, Spain; ^6^ Division of Hepatology and Gene Therapy, Proteomics and BioInformatics Unit, Centre for Applied Medical Research (CIMA), University of Navarra, Pamplona, Spain

**Keywords:** B-cell differentiation, naive B cell, centroblast, centrocyte, memory B cell, quantitative proteomics, transcriptomics integration

## Abstract

Human B-cell differentiation has been extensively investigated on genomic and transcriptomic grounds; however, no studies have accomplished so far detailed analysis of antigen-dependent maturation-associated human B-cell populations from a proteomic perspective. Here, we investigate for the first time the quantitative proteomic profiles of B-cells undergoing antigen-dependent maturation using a label-free LC-MS/MS approach applied on 5 purified B-cell subpopulations (naive, centroblasts, centrocytes, memory and plasma B-cells) from human tonsils (data are available *via* ProteomeXchange with identifier PXD006191). Our results revealed that the actual differences among these B-cell subpopulations are a combination of expression of a few maturation stage-specific proteins within each B-cell subset and maturation-associated changes in relative protein expression levels, which are related with metabolic regulation. The considerable overlap of the proteome of the 5 studied B-cell subsets strengthens the key role of the regulation of the stoichiometry of molecules associated with metabolic regulation and programming, among other signaling cascades (such as antigen recognition and presentation and cell survival) crucial for the transition between each B-cell maturation stage.

## Introduction

The final stages of antigen-dependent human B-cell differentiation leading to (oligo)clonal B-cell expansion and affinity maturation are associated with activation of naive B-cells to their terminal differentiation into antibody-secreting plasma cells (PC) and memory B-cells, in secondary lymphoid tissues (SLT) such as tonsils (1). To initiate this highly dynamic and strictly regulated process, immature B lymphocytes leave the bone marrow and migrate *via* the peripheral blood (PB) to the spleen for maturation into naive B cells, which can then travel to the germinal centers (GC) of SLTs (2, 3). The antigen-dependent maturation starts with the presentation of protein antigens to helper T cells and the production of cytokines. Then, a broad antibody repertoire can be generated in developing B cells by means of somatic recombination. In order to increase the affinity and avidity of antibodies, an affinity maturation process occurs as a result of somatic hypermutation of immunoglobulin (Ig) genes in B cells, giving rise to the selection of strongly binding B cell receptors (BCR) (**Supplementary Figure S1A**) (3, 4).

Within the GC, the dark zone—where proliferation and somatic hypermutation occur—hosts the centroblasts (CB), which further differentiate into smaller centrocytes (CC) that enter the light zone of the GC. Both GC B-cell populations display a considerable overlap in their gene expression profiles, despite showing a clearly distinct morphological appearance (4, 5).

The specific molecular pathway, leading to the generation of memory B-cells following the GC reaction still remain largely unclear (5, 6). Most likely, different factors, such as the affinity of the BCR and the CD40 and CD40L signaling are key elements involved in this process (6). Thus, Smith et al. (7) have proposed a model for memory selection in which memory B-cells and PC are formed from different GC driving pathways, and Scheeren et al. revealed the role of STAT5 in the regulation of memory B-cell differentiation (6). In addition, Kulis et al. (8) through the analysis of the DNA methylome of 5 maturation-associated subpopulations of B-cells (pre-BII cells, naive B- cells, GC B-cells, memory B-cells and PC) showed a dynamic CpG methylation pattern during B-cell maturation, where memory B-cells and bone marrow PC showed the lowest methylation levels.

In recent years, several studies have investigated the genomic, epigenetic and transcriptomic profiles along normal B-cell differentiation (9–15). In turn, most data generated so far about the proteomic profile of B-cells at distinct differentiation stages has been derived from the study of cell lines at different time points after *in vitro* stimulation (e.g. anti-sIgM, lipopolysaccharide) using classical (16, 17) and innovative (18, 19) proteomic strategies. To the best of our knowledge, only one proteomic study (20) analyzed SLT-derived primary GC cells to investigate the similarities between the proteome of mantle cell lymphoma (MCL) cells and normal GC cells. However, the quantitative proteome of SLT-derived non-tumoral human primary B-cells along antigen-dependent B-cell maturation (from naive B-cells to PC and memory B-cells) has not been described so far.

Identification of aberrant protein profiles (related to the splicesome, proteasome, phagosome, HLA molecules, protein synthesis and stability) associated with physiological and pathological conditions such as aging, cancer, auto-immunity, allergy or immunodeficiency is of key relevance for the understanding of the underlying mechanisms involved since proteins are the biochemical effectors in virtually all cellular processes (21). Therefore, the analysis of the proteome of the normal B-cell counterpart of e.g. tumoral B-cells becomes a critical step.

Here, the role of multiple metabolic pathways at different stages of antigen (Ag)-dependent maturation is revealed from the overall (quantitative) global proteome of non-tumoral B-cell populations at different stages of Ag-dependent maturation from naive B-cells to recently generated PCs and memory B-cells from primary non-tumoral tonsils. Furthermore, we integrated the proteomics data set with publicly available transcriptomic data sets to provide a better view and understanding of the normal B-cell maturation process triggered by recognition, and to create a reference map for the identification of altered B-cell-associated protein expression profiles during life and in specific disease conditions.

## Materials and Methods

### Sample Collection

Freshly collected human tonsils were obtained from 5 donors (see **Table 1**) after routine tonsillectomies. In cases A, B, C, and D, tonsils were removed due to more than seven documented throat infections in the year preceding the surgery. In case E, tonsillectomy was performed to improve obstructive sleep apnea. In all cases, informed consent was given by the donor according to the guidelines of the local ethics committee of the University Hospital of Salamanca, in accordance with the Helsinki Declaration of 1975, as revised in 2008.

**Table 1:** Tonsil sample characteristics.

Tonsil	Sex	Age^a^ (years)	Diagnosis	Tonsil-purified cells^b^ (x 10^6^) [%purity]				
				N	CB	CC	M	PC
I	M	6	Sleep apnea	6.9 [95%]	10.4 [95%]	8.5 [95%]	10.5 [90%]	1.7 [75%]
II	F	4	Tonsillitis	15.0 [98%]	13.0 [95%]	16.8 [99.9%]	12.8 [95%]	1.1 [85%]
III	F	26	Tonsillitis	5.7 [98%]	2.1 [98%]	2.1 [99.5%]	4.4 [98%]	<0.1 [91%]
IV	F	20	Tonsillitis	5.2 [93%]	5.5 [96%]	4.5 [99%]	3.3 [93%]	<0.1 [70%]
V	F	17	Tonsillitis	8.0 [98%]	3.0 [93%]	8.9 [99%]	8.3 [97%]	<0.1 [82%]

^a^Age at time of surgery.

^b^By FACS-Aria sorting (Becton/Dickinson Biosciences, San José, CA).

M, male; F, female; N, naive B-cell; CB, centroblast; CC, centrocyte; M, memory B-cell; PC, plasma cell.

The sex and age of the patients, as well as the diagnosis for tonsil surgery, are indicated. Additionally, the number of total purified cells from each tonsil sample is depicted together with the purity percentage of the sorted cells.

Single tonsil cell suspensions were obtained (immediately after surgery) by using conventional mechanical disaggregation procedures (3) in PBS. A minimum of 150 x 10^6^ tonsil cells were stained in parallel in several different tubes (15 min at room temperature (RT) in the darkness) with the following 8-color combination panel of monoclonal antibodies: CD45 conjugated with fluorescein isothiocyanate (CD45-FITC), CD184 conjugated with phycoerythrin (CD184-PE), CD38 conjugated with peridinin chlorophyll protein-Cy5.5 (CD38-PerCPCy5.5), CD10 conjugated with PE- Cy7 (CD10-PECy7), CD20 and CD19 conjugated with APC (CD20-APC, CD19-APC), CD3 conjugated with allophycocyanine-H7 (CD3-APCH7), and CD27 conjugated with Brilliant Violet™ 421 (CD27-BV421) (22). Then, B-cell populations were systematically sorted by FACSAria (BD) at 4°C (cell population gating strategy shown in **Supplementary Figure S2**, sorting purity values shown in **Table 1**) based on the following phenotypes: naive B-cells (CD45^+^, CD184^-^, CD38^-^, CD10^-^, CD19/CD20^+^, CD3^-^, CD27^-^), centroblasts (CD45^+^, CD184^+^, CD38^+^, CD10^+^, CD19/CD20^+^, CD3^-^, CD27^het^), centrocytes (CD45^+^, CD184^-^, CD38^+^, CD10^+^, CD19/CD20^+^, CD3^-^, CD27^het^), memory B-cells (CD45^+^, CD184^-^, CD38^-^, CD10^-^, CD19/CD20^+^, CD3^-^, CD27^+^), and plasma cells (CD45^+^, CD184^-^, CD38^++^, CD10^+^, CD19/CD20^+^, CD3^-^, CD27^++^) (3). Purified cells were immediately processed for protein extraction.

### Protein Extraction

Each cell population (naive B-cells, CB, CC, memory B-cells, and PC) was washed three times with PBS (5 min, 1500 g). After draining off the total PBS volume without disturbing the cell pellet, the lysis buffer (9 M urea, 1 mM activated sodium orthovanadate, 2.5 mM sodium pyrophosphate, 1 mM β-glycerol phosphate, 20 mM HEPES pH 8.0) was added to the cell pellet (15 μL per 1 x 10^6^ cells), followed by sonication on ice (3 bursts of 15 seconds each). Then, samples were centrifuged at maximum speed for 15 min and the supernatant containing the proteins was collected at -80°C until further processing.

### Protein Quantification and SDS-PAGE

Proteins were quantified using the DC™ Protein Assay Kit II, as recommended by the manufacturer. A total of 15 μg of proteins from naive B-cell, CB, CC and memory B-cell samples were separated on a 4-20% gradient SDS-PAGE gel. After electrophoresis, gels were stained in a solution of 0.5% (w/v) Coomassie Brilliant Blue and stored at 4°C in an aqueous solution containing 1% (v/v) acetic acid, until analysis. PC protein samples were processed in solution.

### In-Gel and In-Solution Protein Digestion

Two protein digestion approaches were performed due to PC sample limitations. Proteins from naïve B-cells, CB, CC, and memory B-cells were digested in gel, whereas proteins from PC were digested in solution since the amount of sample was not enough (high difficulty in isolating PC from tonsils due to their low relative numbers) to be processed in the gel. For in-gel protein digestion, each gel lane was cut into five fragments and digested with trypsin following the method of Shevchenko et al. (23) with slight modifications. Briefly, gel pieces were destained with 50% acetonitrile (ACN) and 50 mM ammonium bicarbonate. Protein reduction and alkylation were performed with 10 mM dithiothreitol (DTT) at 56°C for 45 min, and with 55 mM iodoacetamide at RT for 30 min, respectively. Proteins were digested with trypsin (6.25 ng/mL) at 37°C for 18 h. The peptide solution was acidified with 0.1% trifluoroacetic acid and desalted by using C18-Stage-Tips columns (24). The samples were partially dried and stored at −20°C until analysis by LC-MS/MS. For in-solution protein digestion, a total of 4 μg of protein from PC were reduced with 10 mM DTT at RT for 45 min and alkylated as previously indicated. Proteins were then digested with trypsin (1:50 w/w) at 37°C for 18 h and the peptide solution was processed as done for in-gel protein digestion.

### LC-MS/MS Analysis

A nanoUPLC system (nanoAcquity, Waters Corp., Milford, MA) coupled to a LTQ Orbitrap XL mass spectrometer (Thermo Fisher Scientific, San Jose, CA) *via* a nanoelectrospray ion source (Proxeon Biosystems) was used for reversed-phase LC-MS/MS analysis. Peptides were loaded onto a trapping column (Symmetry 300 C18 UPLC Trap Column, 180 μm × 20 mm, 5 μm, Waters Corp.). Peptides were separated on a BEH130 C18 column 75 μm × 200 mm, 1.7 μm (Waters Corp.) equilibrated in 3% ACN and 0.1% formic acid with a linear gradient of 3% to 50% ACN at a flow rate of 300 nL/min over 140 min for in-gel digested proteins (naive B-cell, CB, CC, memory B-cell samples), and 170 min for in-solution digested proteins (PC samples). The nUPLC- LTQ Orbitrap XL was operated in the positive ion mode by applying a data-dependent automatic switch between survey MS scan and tandem mass spectra (MS/MS) acquisition. Survey scans were acquired in the mass range of m/z 400 to 2000 with a 30 000 resolution at m/z 400. The 6 most intense ions with states of 2 and 3 were selected in the ion trap for fragmentation by collision-induced dissociation with normalized energy. Dynamic exclusion was enabled for 30 s.

### Gene Expression Microarrays

Data from the expression profiling of FACS-sorted B-cell subsets from 6 human tonsils on Affymetrix Human Exon 1.0 ST arrays were downloaded from the NCBI’s Gene Expression Omnibus dataset (accession code GSE69033). Raw data was processed as described below.

### Proteomics Analyses

Full downstream analysis was done employing a homemade custom program, using R v.3.4 (25) in the RStudio suite (26) (**Supplementary File 1**).

### Qualitative Protein Expression Profiles

A binary heatmap of absence-presence was generated in order to compare all populations and analyze which patterns were shared by the different replicates. For each population, functional and pathway analyses *via* over-representation tests were done using the clusterProfiler package (27).

### Quantitative Protein Expression Levels

Due to protein retrieval constraints, only four populations (CB, CC, naive, and memory subsets) could be compared for quantitative protein expression levels. Using the Progenesis suite, an ANOVA test with a q-value cutoff of 0.05 followed up by individual T-tests was used to determinate which proteins discriminated among different cell populations. The corresponding heatmap was generated by normalizing each protein by its own z-score among them. Absent proteins in a sample were considered to have zero expression. Hierarchical dendrograms using Euclidean distances were employed for clustering both genes and cell populations.

### Transcriptomics Analyses

To operate with the gene expression profiling and analysis of the different populations of B-cells, the gene expression results of the GSE69033 (13) data set, including six biological replicates of each population, were selected. The mRNA of these cells was hybridized on an Affymetrix Human Exon 1.0 ST Array high-density oligonucleotide microarray. For each gene, an ANOVA test was performed followed by a Bonferroni p-value correction, and those genes with a p-value lower than 0.05 were investigated by a Tukey HSD test with the same cutoff, to determinate which populations could be distinguished.

### Integration of Proteomics and Transcriptomics Datasets

Our proteomics and transcriptomics datasets come from different samples and subjects. To address this issue, a proteomics set and a transcriptomics set for each population were generated including the mean value for each protein or transcript present in all replicates (**Supplementary File 1**). To determine the expression patterns using both data sets, three Circos track plots (28) were generated. Each Circos plot contained line tracks with the quantitative information of the four comparable cell populations; one Circos plot had information of the log10 of our proteomics data (only proteins present in all 5 replicates), the second one for the log10 of the transcriptomic data (only transcripts whose proteins were present in all 6 replicates), and the last one contained the information of the log10 of the direct ratio between the proteomics and transcriptomics datasets. The results of the earlier mentioned ANOVA tests and follow-up tests were represented in attribute plots (29). At the same time, protein/transcript ratios were represented as a heatmap, scaling each ID by its own z-score. Populations were clustered *via* hierarchical dendrograms, using Euclidean distance, while ratios were clustered in four groups using density based clustering of applications with noise clustering *via* the dbscan package (30). ID conversion and coordinate retrieval were done using the biomaRt package (31, 32).

### Quantification Analysis Using Progenesis

Progenesis QI for proteomics v3.0 software (Nonlinear Dynamics, Quayside, Newcastle Upon Tyne, UK) was used for quantification of the multiplexed LC-MduriS data. We compared the B-cell populations by analyzing 5 biological replicates. Specifically, for in-gel digested samples, the Progenesis LC-MS fractionation workflow was performed analyzing 5 fractions from each sample (each fraction corresponded to each band gel sliced during the gel processing). For sample alignment, the sensitivity was set to 5 and MS spectra with intensities > 500 0000, and ions with charges 2-7 were considered for the filtering process. Normalization was applied automatically to all features. Peptide identification was performed using Mascot Search engine (Matrix Science, London, UK) on Proteome Discoverer 1.4. software (Thermo) by searching against the neXtProt database (release December 20, 2016), including the common contaminant sequences (e.g., human keratins, trypsin, BSA). Search parameters were set as follows: carbamidomethylation of cysteine as a fixed modification, oxidation of methionine and acetylation of the protein N-terminus as variable ones, precursor and fragment mass tolerances were set to 10 ppm and 0.6 Da, respectively, and fully tryptic digestion with up to two missed cleavages. False discovery rate (FDR) was set at 1%. Peptide results were imported into the Progenesis QI software, for quantitative analysis and statistical evaluation. Identified peptides were refined removing identifications with a score less than 30, sequence length less than 6 and not human proteins. Peptides assigned to more than one protein were considered conflicting and resolved according to Progenesis guidelines. For reporting peptides and proteins, ANOVA (p ≤ 0.05) and max fold change (≥2) values were calculated.

## Results

### Mapping Ag-Dependent B-Cell Differentiation *via* Characterization of the Global Proteome of Tonsillar B-Cells at Different Maturation Stages

Qualitative evaluation of the global proteome of human B-cells at different stages of maturation was performed on purified naive B-cells, CB, CC, memory B-cells, and PC from 5 primary human tonsils (**Table 1**) as described in the *“Materials and Methods”* section (**Supplementary Figure S1**).

**Figure 1:**
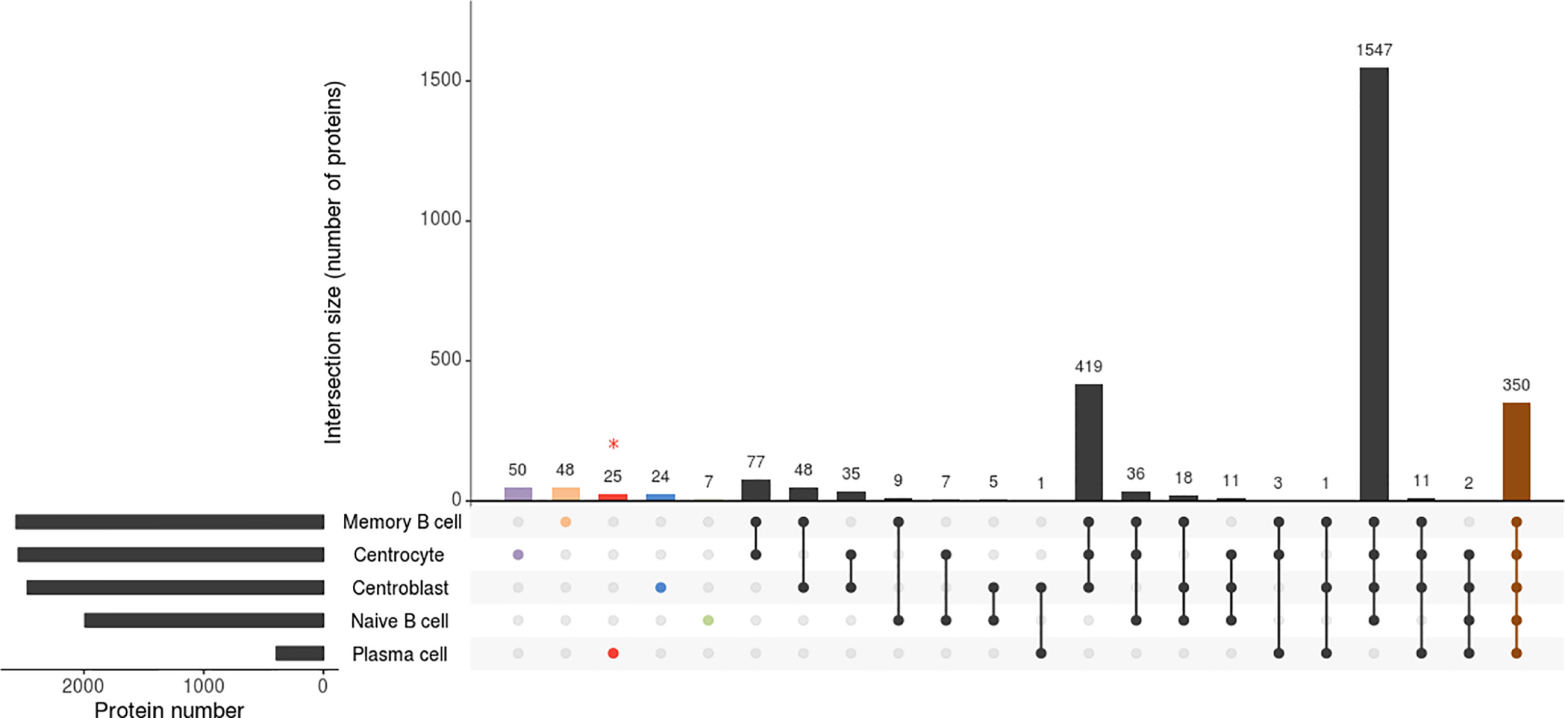
Attribute plot displaying the qualitative proteomic analysis of the 5 B-cell subpopulations (naive B-cells, centroblasts, centrocytes, memory B-cells, and plasma cells). Each column indicates unique protein numbers and corresponds to either a unique population (violet-, orange-, red-, blue- and green-filled dots for centrocytes, memory B cells, plasma cells, centroblasts and naïve B cells, respectively) or a set of populations (black- and brown-filled intersected dots). The bar chart on the bottom left side plots the total number of proteins identified per B-cell population. * The proteomics processing of plasma cells was different due to sample limitations.

Only those proteins that were identified in all B-cell subpopulation replicates of the 5 tonsil samples were considered for further analyses. Overall, similar numbers of distinct proteins were identified for all B-cell subsets analyzed (a total of 1,992 proteins were identified on naive B-cells, 2,472 on CB, 2,548 on CC, and 2,567 on memory B-cells) except for PC (n=393), which is potentially due to the overall lower protein amount obtained from the purified PC samples vs the other 4 B-cell subpopulations (median of 4 vs 15 μg of protein, respectively), due to the very low frequency of PC in tonsils (**Supplementary Table S1**; **Figure 1**). A more detailed analysis of the distribution of these proteins revealed an overlap of 350 proteins among the 5 B-cell subpopulations, up to 1,547 proteins being shared by naive B-cells, CB, CC and memory B-cells (in-gel processed subsets) (**Supplementary Figure S1**).

The binary heatmap in **Supplementary Figure S3** illustrates the clustering of tonsil B-cell subsets based on the presence/absence of proteins (qualitative proteomics characterization). Thus, two main groups were observed; group 1 included only the PC while group 2 included naive B-cells, CB, CC, and memory B-cells. Since such clustering was mainly due to the limited amount of sample processed for PC as discussed above, we further investigated the differences in protein expression profiles of the cell populations included in the second group. Such analysis showed that, among the 4 B-cell populations in the second group, naive B-cells were those showing a clearly distinct proteome, with clearly lower numbers of proteins identified, and suggesting that the overall number of proteins expressed increases as B-cell differentiation progresses from naive to memory B-cells. Of note, proteins exclusively expressed in GC B-cells (33) (**Supplementary Table S2**), mostly included proteins involved in DNA and RNA synthesis (RNA polymerase, MED21; RNA helicase, DDX47; DNA replication complex, GINS3; transcription, GTF2A2; translation, EIF4G2; initiation factors; nuclear pore glycoprotein, NUP62; and histone, HIST2H2BC). In turn, the proteins expressed solely in GC B- cells (i.e. CB and CC) laid in the expression of 24 of 2,472 (1%) specific proteins (**Figure 1**; **Supplementary Table S2**) in CB (proteins affecting B-cell receptor signaling such as VAV1, VAV2, GTPase RAP1GDS1; proteins involved in transcription process and histone acetylation such as BRD2 and in cell apoptosis process such as FADD) and 50 of 2,548 (2%) in CC (**Figure 1**; **Supplementary Table S2**) (e.g. NR3C1, BCL7A, PEG10, RGS13). Likewise, the investigation of naive B-cells displayed 7 of 1,992 (0.4%) proteins uniquely identified in this cellular subset (RNASEH2C, MRPL21, PURB, NDUFA3, HBQ1, PCBD2, and C9orf64) (**Figure 1**; **Supplementary Table S2**). Interestingly, the expression of specific proteins may determine the fate of the B-cells from the GC to differentiate into memory B-cells or PC. The qualitative comparison of the protein profiles of both subpopulations (i.e. memory B-cells and PC) (**Supplementary Table S1**) displayed that 29 proteins of the PC proteome were not expressed by memory B-cells, 25 of 393 (6%) of them were exclusively expressed in PC. These 29 PC-specific proteins included specific Ig molecules (IGKV3-11, IGKV3-15, IGHD, IGLC3, and JCHAIN), cytochrome C oxidases (COX6B1, COX7C), regulatory and transcription factors (IRF4, GTF2A1, LZTFL1), and the Abl interactor (ABL1) among other proteins. In addition, two of the remaining proteins were detected in both cell subsets but in different isoforms - isoform 1 (memory B-cells) and isoforms 5 and 2 (PC) for ADAR and PRKCAB proteins, respectively-. In contrast, 48 of 2,567 (2%) proteins were exclusively expressed by memory B- cells and not detected in PC (**Supplementary Table S2**), including the EBF1, BCL2L13, LRBA, IRF9, ITGB1, NMI, MRPL55, and THEMIS2 proteins.

### Quantitative Maturation-Associated Protein Profiles From Naive to GC and Memory B-Cells

For quantitative protein analyses, a restriction criterion was established when a protein presented a value=0 for ≥1 of the 5 sample replicates investigated within each B-cell subpopulation; in such case, a value=0 was also assigned to the other replicates for that subpopulation (i.e. following the criteria established above for the qualitative analysis). However, the protein was not removed from the analysis as 0 has a quantitative meaning (absence of protein expression). In contrast, whenever a protein was “absent” across all B-cell populations analyzed, that protein was removed from further analyses. Thus, only differentially expressed proteins (ANOVA p-value<0.05) were included in the quantitative analysis for a total of 753 proteins expressed at significantly different levels (p-value<0.05) among the distinct B-cell subsets (**Supplementary Table S3**). Of note, due to the limited protein amount of PC protein samples, this B-cell subpopulation was not included in the quantitative proteome analyses that were thereby, exclusively performed on naive B-cells, CB, CC, and memory B-cells.

The quantitative proteomics data collected in **Supplementary Table S3** was the basis for the principal component analysis (PCA) shown in **Supplementary Figure S4**, which revealed grouping of the B-cell subpopulations according to the relative protein abundances with a clearly higher separation between naive B-cells and the other three B-cell populations. In turn, CB and CC clustered closely together, while memory B-cells appeared to be closer to naive B-cells.

The distribution of the 753 differentially expressed proteins across the distinct human chromosomes and the different B-cell subpopulations are depicted in **Supplementary Figure S5** (panels **A**, **B**, **C**). Of note, highly similar protein expression patterns were detected at the chromosome level for the GC and memory B-cells in contrast to naive B-cells, the latter showing lower expression levels for specific proteins encoded on chromosomes 1-4, 6, 8-9, 11, 14-15, 22 and X. Regarding the remaining chromosomes, protein expression profiles were similar among the four maturation-associated B-cell subsets analyzed, except for chromosome 11, for which naive B-cells and CB on one side, and CC and memory B-cells on the other side, were closer to each other.

**Figure 2:**
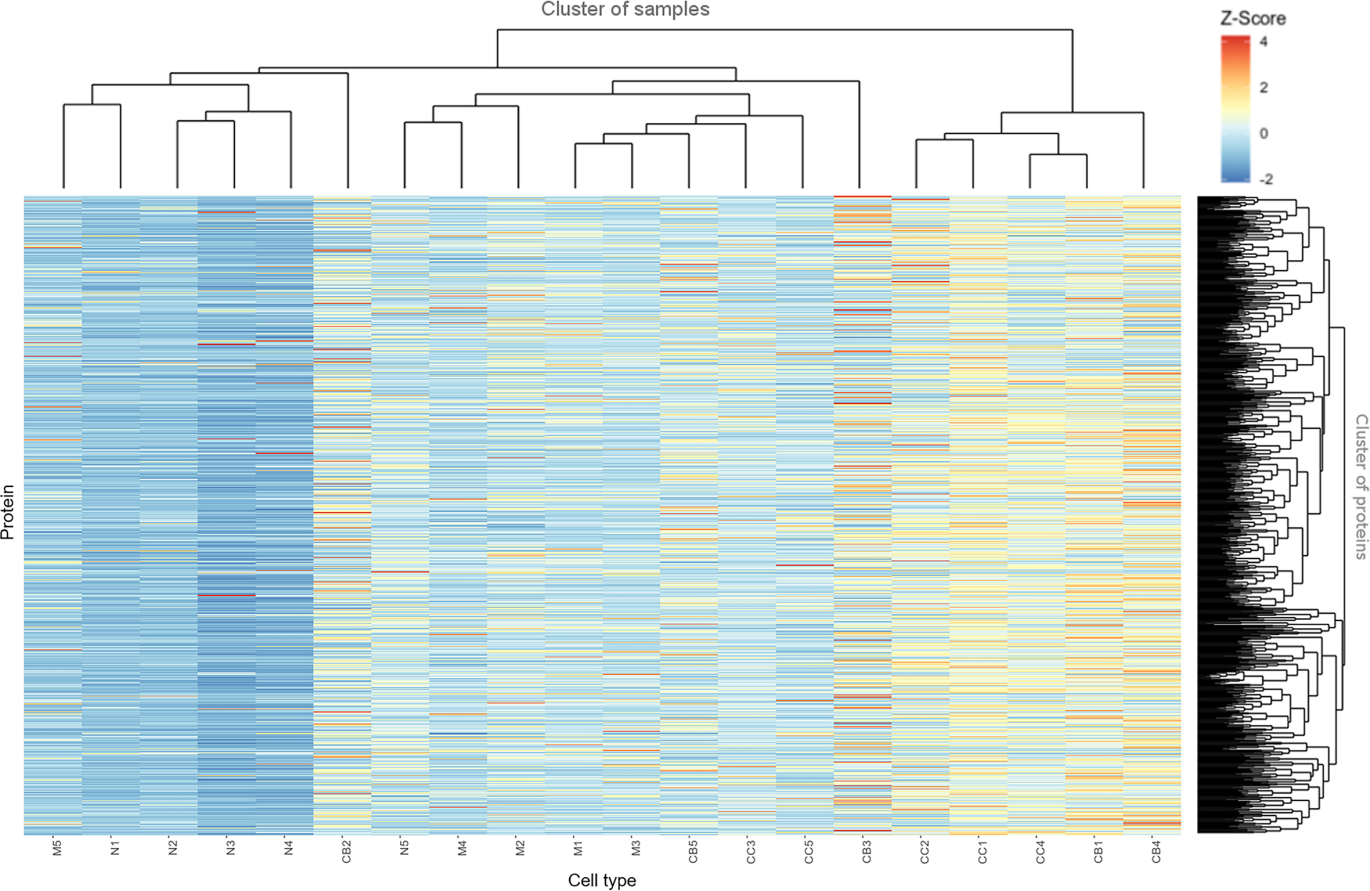
Heatmap based on Z-score values for the proteins identified and quantitatively measured in naive B-cells (N), centroblasts (CB), centrocytes (CC) and memory B-cells (M). The color gradually changed from 4 (red) to 1 (yellow) and -2 (blue).

**Figure 2** depicts a hierarchical clustering analysis of the levels of these proteins differentially expressed among the distinct B-cell populations (naive B-cells, CB, CC, memory B-cells) (n=753) for each tonsil (1–5) sample analyzed. Overall, a high similarity was observed for all samples between CB and CC on one side, and to a less extent also, between naive and memory B-cells on the other side (except for two outliers, M5 and CB2). As discussed already above, this quantitative heatmap (together with that of **Supplementary Figure S3**) showed that the lowest protein expression values corresponded to the naive B-cells (coded as blue, Z-score values<0) whereas the highest ones corresponded to both CC and CB (coded as orange and red, respectively; Z-score values ≥2). Protein expression profiles of memory B-cells were more heterogeneous, including proteins presenting high expression levels and others being absent or expressed at low levels.

The distribution per cell population of the proteins showing the highest expression levels was as follows: 363 of 753 (48%) proteins were most strongly expressed in CB; 271 of 753 (36%) in CC; 97 of 753 (13%) in memory B-cells; and 22 of 753 (3%) were most strongly expressed in naive B-cells (as illustrated in **Supplementary Figure S6**). Conversely, the lowest expression levels for most proteins corresponded to the less differentiated cells (naive B-cells) - 608 of 753 (81%) proteins -, followed by memory B-cells – 92 of 753 (12%) -, and both CC and CB – 29 of 753 (4%) and 24 of 753 (3%) -, respectively (**Supplementary Table S3**).

Pairwise comparisons (1 vs 1) between the different B-cell subsets (**Supplementary Table S3**) showed (summarized results in **Supplementary Table S4** and **Supplementary Figure S7**) proteins with significantly different relative abundances between each pair of B-cell populations (p-value<0.05). Once again, naive B-cells were those with lower numbers of proteins being over-expressed (<10% of all differentially expressed proteins). Of note, CB and CC showed a homogeneous distribution (60-40%, respectively) indicating the great similarity between both B-cell populations. Additionally, proteins associated with memory B-cells were found to be under-expressed in all pair-wise comparisons, except vs naive B-cells.

### Functional Evaluation of the Differential Protein Profiles Identified Across Different Maturation-Associated B-Cell Populations

Functional enrichment screening for those proteins differentially expressed across the distinct B-cell populations in the 5 tonsil samples analyzed (**Supplementary Table S5**) revealed that those 350 proteins which were systematically detected in all B-cell subsets (**Supplementary Table S1**) were annotated in 56 functional clusters highly enriched in general cell functions (e.g. actin and tubulin binding, biosynthesis, proteolysis, cell-cell adhesion, RNA processing and glucose metabolism).

Further analyses of the functional pathways associated with the individual B-cell subsets (**Supplementary Figure S8**; **Supplementary Table S5**) showed that proteins that were exclusively identified in naive B-cells participated in a few pathways (e.g. ribosome, metabolic pathways, and DNA replication) also common to the other B-cell subsets. In turn, memory B-cell proteins were related to a greater number of pathways, particularly the protein processing (i.e. synthesis, PTMs, stability, degradation), antigen processing and presentation, and JAK-STAT signaling pathways. Specific proteins expressed by CB and CC were related to Fc-gamma-R-mediated phagocytosis and chemokine signaling pathways. In turn, CB and memory subsets shared (specific) proteins belonging to the Toll-like receptor, TGF-beta, WNT, cAMP, IL17, Fc epsilon R, TNF and cell cycle signaling pathways. Likewise, the ERBB, PI3K-AKT, MAPK, and mTOR signaling pathways included proteins exclusively identified on CC and memory B-cells. Regarding PC-specific proteins, these were mostly involved in protein export, spliceosome, and metabolic pathways. Overall, the EGFR tyrosine kinase inhibitor resistance, DNA replication, MAPK signaling, antigen processing and presentation, ribosome, protein export, spliceosome and metabolic pathways were those most differentially represented across the naive, CB, CC, memory and PC proteomes.

Protein enrichment within these networks revealed the more significant modules (**Supplementary Figure S9A**, **Supplementary Table S6**) and pathways (**Supplementary Figure S9B**, **Supplementary Table S6**) involved. Thus, the module with a greater number of genes/proteins detected in all B-cell subpopulations related to ribosome/protein synthesis, also associated with the spliceosome network in the pathway analysis. Of note, the citrate cycle was the most significant pathway in PC.

### Quantitative Protein Differences During B-Cell Differentiation

**Figure 3:**
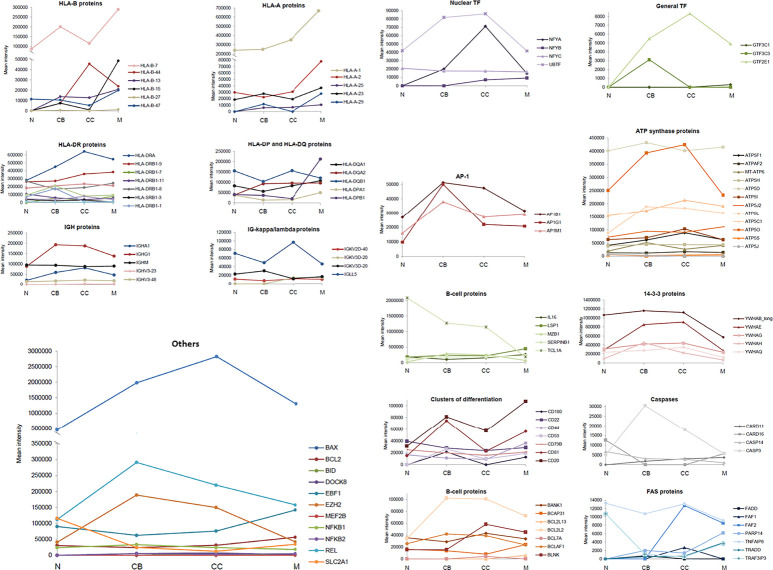
Expression of specific groups of proteins across the different maturation-associated B-cell populations analyzed. The mean intensity obtained in the mass spectrometry analysis of the 5 replicates for each B-cell population was calculated and represented by family groups. *N*, naive B-cells; *CB*, centroblasts; *CC*, centrocytes; *M*, memory B-cells.

The variation in the (quantitative) levels of specific proteins throughout the four maturation-associated B-cell populations analyzed (i.e. naive B-cells, CB, CC, memory B-cells) provided interesting insights into the B-cell differentiation process. Relevant protein groups for B-cell development, functioning and survival are depicted in **Figure 3** and listed below. Findings are further explained in the *Discussion* section.

*HLA proteins, responsible for the regulation of the immune system*. In general, HLA-A proteins showed an increased expression in CB and memory B-cells with decreased values (even absent) in naive B-cells and CC. HLA-A-1 was significantly over-expressed in memory B-cells. Regarding HLA-B proteins, their expression progressively increased with higher B-cell differentiation from naive to memory B-cells, except for HLA-B-44. In turn, the HLA-DP, HLA-DQ and HLA-DR proteins showed a similar expression pattern, with a transient reduction in CB followed by a progressive recovery up to the memory B-cell subset; HLA-DOA displayed significantly higher expression levels in naive and memory B-cells.

*Ig, serving as antigen receptors or neutralizing agents against specific antigens*. Concerning Ig, no significant differences were observed for the IGHV as well as IGHM proteins. However, IGHA and IGHG1 displayed higher expression levels in GC B-cells (i.e. CB and CC). Regarding the Ig kappa/lambda light chains, the greatest differences among B-cell subsets were observed for the IGLL5 protein whose expression levels were decreased in CB and memory B-cells, while increased in naive B-cells and CC.

*14-3-3 proteins, playing an isoform-specific role in class switch recombination*. The expression profile of 14-3-3 proteins was constant across all 4 B-cell populations with higher levels in GC B-cells (CB and CC), and lower levels in naive and memory B-cells.

*Caspases, for cell cycle regulation*. Caspases and caspase-related proteins showed an unequal distribution along B-cell differentiation: some of these proteins showed low levels in CB and CC (CARD16, CASP14), another displayed increasing expression levels from naive to memory B-cells (CARD11), and another one had clear higher expression levels in the CB (CASP3).

*B-cell associated proteins and adhesion molecules*. The expression of these membrane proteins was characterized by either high levels in CB and memory subsets (CD20, CD81, CD53, CD180) or uniform expression levels across all 4 B-cell subsets with a slight increase among naive B-cells (CD22, CD44, CD79B).

*FAS-associated proteins, involved in apoptosis*. Interestingly, Fas-associated proteins were absent in naive B-cells (FADD, FAF1, FAF2, PARP14, TRADD), except for TRAF3IP3 and TNFAIP8, which presented the highest levels in this B-cell subset. For the TRADD and PARP14 proteins, memory B-cells were those showing the highest expression values.

*Transcription factors (TF)*. Expression of TF was increased in GC B-cells (CB and CC) compared to naive and memory B-cells, both as regards the nuclear and overall TFs identified to be positively expressed. The same distribution pattern was found for the AP-1 proteins.

*ATP synthase proteins, to catalyze the cell energy production*. No significant differences were observed for these proteins among naive, CB, CC and memory B-cells, except for ATP5O, which almost doubled its expression in CC (vs. naïve and memory B-cells).

*“Missing Proteins” and their patterns across B-cell subpopulations*. The Human Proteome Project (HPP) has identified a set of proteins called “missing proteins” in based the reported evidence of these proteins (34). These missing proteins are classified by HPP in 5 groups called PE1 (evidence at protein level), PE2 (evidence at transcript level), PE3 (inferred from homology), PE4 (predicted), PE5 (uncertain). Then, complementary study to quantitative protein expression levels was evidence of protein existence (PE) classification (way to classify the so-called “Missing Proteins”). Here, it is explored only from the protein datasets (mean protein expression value) which identified protein candidates on groups PE1, PE2, PE5. Thus, 606 proteins have been correlated with a transcriptomics counterpart in the expression microarray, where 599 are include in PE1, one in PE2 and 6 in PE5. In the case of PE2 protein, RGS13 (with evidence at transcript level) is involved in signal transduction; while the six proteins (NCF1B, HSP90AB3P, HIST2HBC, SNX29P2, RPL0P6, POTEKP) in group PE5 are involved in stress response, DNA-binding, phosphatidylinositol binding, ribosomal protein and ATP binding (**Supplementary Table S7**). Within this group of PE5 proteins, the proteins HSP90AB3P, HIST2HBC and RPLP0P6, related with DNA-binding, stress response and ribosomal functions, show a differential protein profile during B-cell differentiation, where low levels are observed in naive B-cells and memory B-cells and progressive level expression increase is depicted for CB, whereas for CC there is a progressive decrease. Regarding the proteins NCF1B and SNX29P2, which are phosphatidylinositol binding proteins, present a similar pattern a similar profile, with progressive increasing of its expression level from naive B-cells to CC, and low levels in memory B-cells. Finally, POTEKP presents a completely different pattern from the other ones, with low levels in naive B-cell, increasing pattern in CB and memory B-cell and decrease profile in CC (**Supplementary Figure S10**).

*Other proteins*. IL16, LSP1, MZB1, SERPINB1, BCAP31, and BCL2L13 showed higher expression levels on memory B-cells vs all other subsets. In contrast, the highest expression levels for TCL1A were found in naive B-cells, gradually decreasing down to the memory B-cells compartment. Also, BCL2L2 presented the lowest expression levels among naive B-cells whereas the highest ones were detected in CB, CC and, particularly memory B-cells. EBF1 was expressed at slightly higher levels in CC vs CB, with the highest levels being observed on memory B-cells. In turn, REL, EZH2, and BAX presented the lowest expression levels in naive B-cells and high expression levels in CB. Regarding the expression level of BCL2, the memory subset displayed the highest values of the quantitative expression.

### Correlation Between the Transcriptome and the Proteome of Distinct Maturation-Associated B-Cell Populations

The results of previously reported transcriptomics analyses, performed on the same maturation-associated B-cell populations, following identical processing and purification strategies (13), were compared with our proteomics results. A total of 6 tonsils were analyzed for transcriptomics and the resulting values (after processing transcriptomics data) were averaged as performed also for the proteomics data of this study.

First, the global list of genes contained in the transcriptomics platform, together with the differentially expressed genes (**Supplementary Table S8**) and their correspondence to differentially expressed proteins (ANOVA p-value<0.05) detected by LC-MS/MS was integrated and evaluated. Results of such comparative analyses (**Supplementary Figure S7A**) revealed a greater number of molecules differentially expressed by transcriptomics than those detected in the proteomics analysis (**Supplementary Figure S7B**), which could have been expected due to the total genome coverage of the transcriptomics platform vs the quantitative proteomics dataset. Thus, only 6% (201 of 3,241) of those transcripts that were found to be differentially expressed were associated also with a significantly differentially expressed (translated) protein. Additionally, a greater number of differentially expressed genes between naive B-cells and GC B-cells was found (2,826 of 3,241 genes; 87%). By contrast, naive and memory B-cells were more similar at the transcriptomic level showing only 450 of 3,241 (14%) genes differentially expressed between them. As done for the proteomics dataset, a representative cluster plot was generated to evaluate the distribution of the genes expressed in the different B-cell populations per chromosome (**Supplementary Figure S5B**).

**Figure 4:**
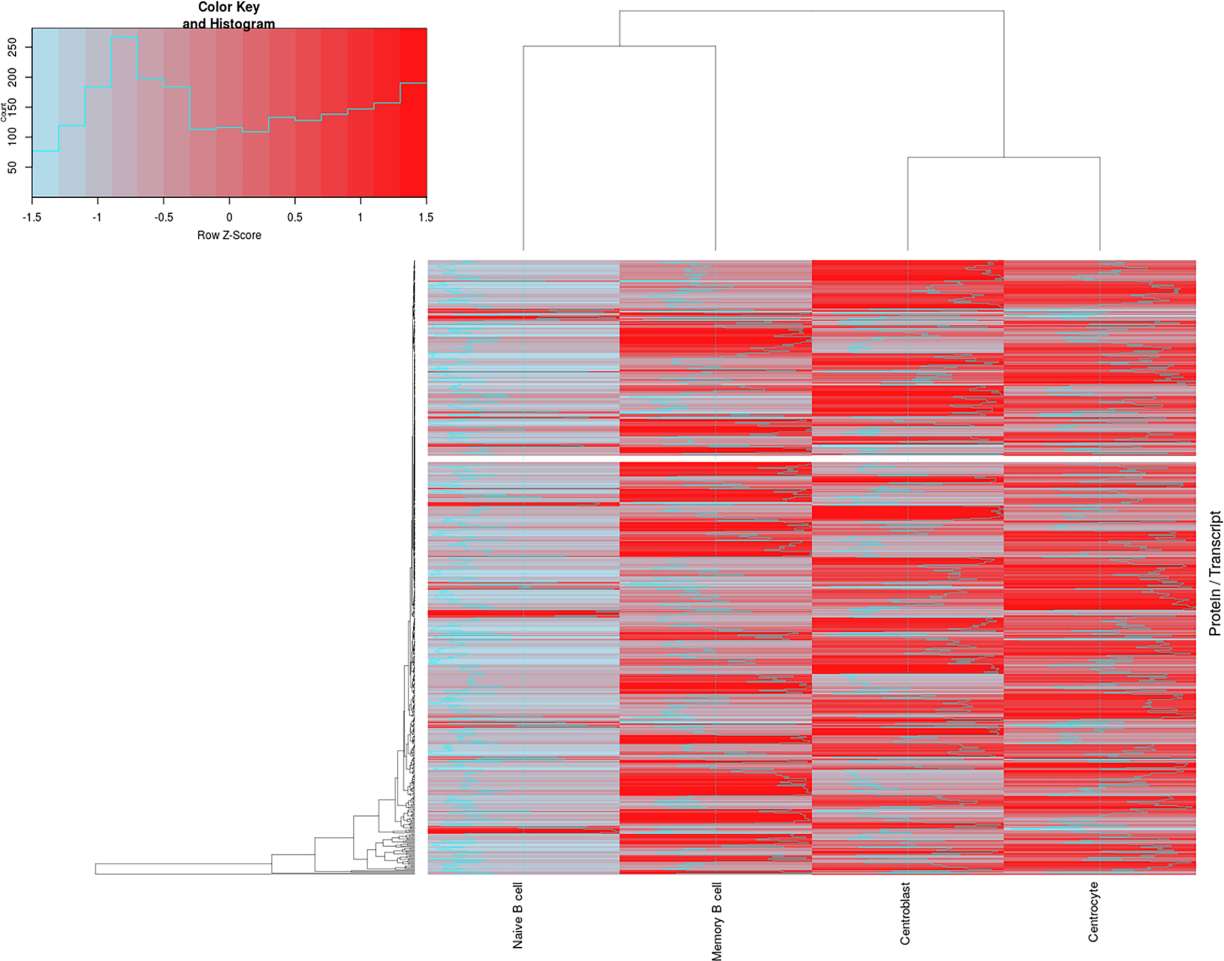
Correlation between the proteomics and transcriptomics datasets for the distinct maturation-associated B-cell populations analyzed. The figure depicts a total of 569 genes/proteins detected by both approaches and the corresponding hierarchical clustering analysis for the 4 B-cell subpopulations (naive B-cells, centroblast, centrocyte and memory B-cells) investigated. Z-score values assuming the differences of proteomics/transcriptomics correlations are color-coded (blue for low P/T ratios and red for high P/T ratios). Lists of genes/proteins, as well as the ratio values, are shown in **Supplementary Table S9**.

To evaluate the correlation between transcriptomics and proteomics datasets for the different B- cell populations analyzed (naive, CB, CC, and memory), an updated version of the R-script tool previously described by our group (35) for integrating both sets of data was used. After calculating the corresponding ratios between proteomics and transcriptomics expression values (**Supplementary Table S9**), the results revealed higher protein/transcript ratios for CB, CC and memory B-cells than for the naive B-cells (**Figure 4**). A total of 569 paired genes/proteins were present in both datasets and therefore compared (expressed genes not detected by the proteomics approach were not included in this correlation analysis, **Supplementary Table S8**). This hierarchical clustering analysis (**Figure 4**) revealed two main groups of cells: group 1 consisting of naive and memory B-cells; and group 2, that included GC (CB and CC) B-cells. These data are also represented in **Supplementary Figure S5C** displaying the differences among the distinct B-cell subpopulations at the gene/protein levels per chromosome (i.e. protein-encoding genes in chromosomes 1, 4-6, 9, 15-16, 19, 22). Also, **Supplementary Table S10** and **Supplementary Figure S11** display five major protein clusters (obtained after self-organizing map (SOM) analysis from data collected in **Supplementary Table S3**) representing once more the protein expression dynamics across the distinct B-cell subpopulations. For example: SRSF2 and PDIA4 proteins from cluster 1 displayed higher gene/protein ratios at CC population, whereas proteins from cluster 5 (TRAF3IP3, CPOX, TPK1, S100A6, DCTD, LGMN) depicted lower ratio levels in the same population.

### Evaluation of the Expression of Proteins Specific for One or More Maturation-Associated B-Cell Populations by Multi-color Flow Cytometry (FCM)

The expression patterns of a subset of 11 differentially expressed proteins across the 4 B-cell populations analyzed (i.e. naive B-cells, CB, CC, and memory B-cells) was also quantitatively evaluated by FCM on the B-cell surface membrane (CD20, CD22, CD44, CD45, CD54, CD71, CD74, CD79B, CD81, and CD98) or at the intracellular levels also TCL1A. Overall, highly similar expression patterns were found for around half of these proteins (511; 45%) (**Supplementary Figure S12**): CD44, CD54, CD71, CD74, and TCL1A. Of note, FCM analysis confirmed our previous results about the expression levels of the TCL1A protein, which decreased with increasing B-cell differentiation from naive to GC and memory B-cells.

## Discussion

Human B-cell differentiation has been extensively investigated on genomics and transcriptomics grounds (12, 15, 35), providing relevant data about those genes (and their modifications) and transcripts that regulate B-cell differentiation. Nevertheless, no studies have addressed so far, a detailed analysis of cells at different Ag-dependent human B-cell maturation stages from a quantitative and differential intracellular signaling proteomic perspective. Despite genomic information is highly relevant to understand the molecular basis of B-cell differentiation, the dynamics of this process can only be fully understood by also exploring and integrating the corresponding transcriptome and proteome information for each maturation stage (33, 34, 36, 37). Specifically, the main level of information required to understand the functioning of cells is the global analysis of proteins (i.e. key functional components of the cell) (38). However, the accomplishment of this examination is complex due to the amino acids features, the protein modifications and degradation, and the intricate and variable signaling networks in which proteins are involved (37).

Here, the quantitative proteomic profiles of human B-cells undergoing Ag-dependent maturation in tonsils have been described, depicting the intracellular pathways involved and the correlation with transcriptomics data. Thus, 5 different B-cell subpopulations at distinct stages of maturation (naive B-cells, CB, CC, memory B-cells, and PC) were highly purified from 5 human tonsils and proteomics-profiled by using a label-free quantitative LC-MS/MS approach. Qualitative analyses were performed on all 5 B-cell subsets, providing insights into their proteome maps that contributed to the understanding of how each B-cell subset gives rise to the next one. Quantitative proteomics comparisons shed further light on those differentially significantly expressed proteins, which may influence the switch among the studied B-cell maturation stages. Overall, our results showed a high overlap of the proteome of the 5 B-cell populations. Focusing on naive B-cells, CB, CC and memory B-cells, which were processed in a fully comparable way, this overlapping involved up to 1,897 proteins, which constituted 95% of proteins expressed by naive B-cells and ~75% of proteins detected in CB, CC, and memory B-cells. As might be expected, these proteins were typically involved in general cell functions such as actin and tubulin binding, biosynthesis, proteolysis, cell-cell adhesion, RNA processing and glucose metabolism. Despite the high intersection among the proteomes of B-cells at different maturation stages, specific proteins were also identified within each B-cell subset. Thus, GC-specific proteins included MED21, DDX47, GINS3, GTF2A2, EIF4G2, NUP62, and HIST2HBC, found in both the CB and CC proteomes, which are associated with FC-gamma-R-mediated phagocytosis and chemokine signaling pathways. In turn, while CB expressed VAV1, VAV2, RAP1GDS1, BRD2, and FADD among other proteins, CC exclusively expressed NR3C1, BCL7A, PEG10, and RGS13 within a total group of 50 unique proteins in the absence of the above listed CB-specific proteins. Of note, RGS13, whose expression was restricted to CC, has an important role in chemotaxis and the limitation of the expansion of GC cells (39). In turn, the expression of the VAV1/2 proteins restricted to CB is crucial for PC development and secretory Ig production (40), suggesting that PC might originate from a subset of CB-cells that up-regulate VAV1 and VAV2 expression leading to the induction of PC formation. In turn, naive B-cells presented only 7 exclusive proteins (i.e. RNASEH2C, MRPL21, PURB, NDUFA3, HBQ1, PCBD2 and C9orf64) involved in ribosome formation, metabolic pathways, and DNA replication.

At present, the precise proteins that are associated with differentiation of GC B-cells to memory B-cells vs PC still remain largely to be identified (41). Overall, our results showed 29 PC-specific proteins that were not identified in paired memory B-cells. These proteins were related to Ig (IGKV3-11, IGKV3-15, IGHD, IGLC3 and JCHAIN), cytochrome C oxidases (COX6B1, COX7C), regulatory and transcription factors (IRF4, GTF2A1, LZTFL1), and the Abl interactor ABL1. From these proteins, IRF4 has been claimed to define Ig-secreting PC by De Silva et al. (4, 42), being its expression associated with protein networks related to protein export, the spliceosome, and metabolic pathways among which the citric acid cycle network is the most significant. This, together with increased expression of cytochrome C oxidases in PC vs GC and memory B-cells might reflect the high energy consumption of PC, probably required because of their high Ig-secreting functionality. In parallel, high expression of multiple transcription factors discloses a unique increase in gene expression in these cells affecting previously silenced genes (i.e. a new transcriptional cell program). Finally, overexpression of ABL1 confirms previous observations by Li et al. (43) which revealed the role of this tyrosine kinase on PC survival.

In turn, the EBF1, BCL2L13, LRBA, IRF9, ITGB1, NMI, MRPL55 and THEMIS2 proteins were exclusively expressed in memory B-cells. Among others, these memory B-cells linked proteins involved in metabolic pathways, antigen processing and presentation, and the JAK-STAT signaling pathway. Thus, LRBA promotes proliferation, clonal expansion and cell survival of antibody-secreting cells and its deficiency has been demonstrated to reduce proliferation (44, 45). In turn, high levels of ITGB1 (also known as CD29) have been detected in memory B-cells (46). Of note, THEMIS2 was exclusively identified in the memory B-cell compartment, despite it seems to be not required for B-cell development (47).

Overall, naive B-cells emerged as the less differentiated cells of all B-cell subsets analyzed as for its protein expression profile strongly overlapped with that of the other B-cell populations, which showed greater proteomic diversity. However, qualitative differences existed for only a limited number of all expressed proteins. In contrast, more differentially expressed proteins were identified when the relative quantity of each protein across the distinct B-cell differentiation stages was considered. Additionally, quantitative analysis depicted 753 proteins to be differentially expressed in naïve B-cells, CB, CC, and memory B-cells. Interestingly, for these proteins, similar amounts and expression patterns were found for CB and CC on one side, and for naive and memory B-cells on the other side, with the lowest protein expression values being detected in naive B-cells further supporting their less differentiated nature. In contrast, the highest levels of protein expression were found for GC B-cells depicting the high functionality and protein turnover characteristic of these cells (2). Subsequent pair-wise comparisons between each B-cell population and both the previous and/or subsequent maturation stages confirmed the highly similar and homogeneous (quantitative) proteomics profile of CB and CC (2). By contrast, proteins of naive B-cells were under-expressed vs all other maturation stages.

More detailed analysis of those specific protein groups differentially expressed across the different maturation-associated B-cell populations revealed new insights into their functional proteomics throughout B-cell maturation. Thus, for the HLA-associated proteins, significantly high expression levels of HLA-DO were found in naive and memory B-cells vs GC cells, confirming previous observations by others studies (2, 48). In contrast, expression of the HLA-DR and CD74 molecules was higher within GC cells compared with the other two B-cell populations. These results support those of Chalouni et al. suggesting that when a specific Ag encounters naive B-cells, synthesis of new HLA II molecules is initiated to favor CB selection (48). Within the HLA I, expression of HLA-A and HLA-B proteins progressively increased as the B-cell maturation process advanced, except for HLA-B44, which would support the greater endogenous Ag-peptide presenting capacity of long-lived PC and memory B-cells vs short-lived naive B-cells. Regarding Ig molecules, two main expression patterns were observed: i) no differences among the B-cell subsets were found for IGHM and IGHV, while ii) greater expression values were found for IGHA, IGHG, Igλ, Igκ in GC B-cells vs both naive and memory B lymphocytes. Whereas the production of the mu and variable heavy chains of the Ig starts in the bone marrow, production of IgG and IgA requires class switch recombination in the GC, which likely explains our observations (49). In line with these findings, the 14-3-3 proteins involved in class-switch recombination expressed also significantly higher levels in CB and CC B-cells as also previously demonstrated by Xu et al. (50).

Interestingly, a relatively high number of differentially expressed proteins play distinct roles in apoptosis and/or survival of B-cells. For instance the CARD11 scaffold protein showed increasing levels from naive B-cells (lacking CARD11 expression) to memory B-cells, this protein being altered in a spectrum of diffuse large B-cell lymphomas (DLBCL) (51), mainly those derived from GC (GCB-DLBCL) and activated B-cells (ABC-DLBCL) (52), where it plays a key role in activating the NF-kB pathway to induce the B-cell proliferation and the proliferation vs death checkpoint in activated B-cells (52). TRADD also activates this pathway (53) and it was found to be overexpressed in memory B-cells vs less differentiated mature B-cells; which may be very interesting from a pathogenic point of view. Conversely, CASP3, a key regulator of Fas-mediated cell death in mature peripheral B-cells (54), was highly expressed in GC cells, particularly in CB (55). Of note, most Fas-related proteins were missing in naive B-cells, which might be due to the need of these cells to expand for continuing their B-cell differentiation and ensuring antibody production.

Study of missing proteins patterns shows expression profiles in accordance with the processes that occur along the cellular differentiation of the B-lymphocyte. High levels of DNA binding protein in CB are related to mutation for function receptor generation and cell proliferation, and this type of proteins decreases its levels as B cells differentiate into CC and memory B-cells. A similar pattern was observed for stress response and ribosomal proteins, since CB presented high expression levels due to the changing environment in which they are found. Regarding phosphatidylinositol binding protein, high levels were observed in CC, correlating with the active cell signaling due to antigen presentation required at this differentiation stage. Finally, ATP-binding protein presented a higher relative expression in memory B-cells compared to others, which correlated with the vesicle transport processes occurring in the immune synapse for antigen presentation in MHC molecules to T lymphocyte (56–61). Regarding other B-cell related proteins, distinct differentiation-associated profiles were observed. Thus, while CD22, CD44, and CD79B showed similar expression values throughout the B-cell maturation, other markers such as TCL1A, CD20, CD53, CD81 and CD80 varied substantially. Accordingly, expression of TCL1A was detected at the highest levels in the first stages of maturation (e.g. naive B-cells), it was reduced upon GC development and silenced in long-lived memory B-cells, as previously shown also by others (62). In contrast, IL16 protein levels peaked in memory B-cells and the highest CD20, CD53, CD81, and CD180 protein amounts were observed in CB and memory B-cells. Of note, expression of CD20 correlated with the expression of both HLA-A and HLA–B molecules, and CD81, which could reflect the well-known role of CD20-HLA-I and CD20-CD81 interactions required for the regulation of cell cycle progression in B-cells (63). Similarly, NFKB1 and NFKB2, two proteins that play an important role in B-cell activation and GC formation, were both detected at higher levels in CB and CC vs naive and memory B-cells (4). In turn, BCL2 was highly expressed in memory B-cells, supporting its role in the development and survival of the long-lived B-cell subset. In line with these observations, the expression of the BAX and BID pro-apoptotic proteins was higher in CB vs both naive and memory B-cells, respectively (64). Finally, the expression of EZH2 was increased in both CB and CC GC B-cells, their levels decreasing thereafter when the cells leave the GC (4).

In summary, our results provide a first map of the proteome of Ag-dependent B-cell maturation in tonsils supporting the value of quantitative proteomics data, which provides more information than that of qualitative proteomics solely based on the presence vs absence of the proteins. In addition, we show that along the differentiation of mature B-cells, the naive B-cell compartment typically shows expression of fewer proteins usually at lower levels, most of which are shared by the immature GC and memory B-cells. Of note, a highly similar proteomics profile was found for CB and CC while memory B-cells more closely mimicked naive B-cells despite both cell compartments are at the greatest distance B-cell maturation process.

## Data Availability Statement

The full mass spectrometry proteomics data reported in this paper has been deposited to ProteomeXchange Consortium via the PRIDE (65) partner repository with the dataset identifier PXD006191.

## Author Contributions

Conceptualization, AO, MF, and PD. Methodology, PD, MP-A, MB, MA, RMD, EB, SM-G, PB, FE, RM-R, VS, AL-V, PJ-V, and KD. Software, RG-V. Validation, PD and RG-V. Formal analysis, PD and RG-V. Investigation, PD. Resources, MF, AO, JA, RG, SSC, MP-A, KD, and FE. Writing—original draft, PD. Writing—review and editing. RM-R, MF, and AO. Visualization, PD, RG-V, VS, AL-V, and PJ-V. Supervision, MF and AO Funding acquisition, MF and AO. All authors contributed to the article and approved the submitted version.

## Funding

We gratefully acknowledge financial support from the Spanish Health Institute Carlos III (ISCIII) for the grants: FIS PI14/01538, FIS PI17/01930 and CB16/12/00400. We also acknowledge Fondos FEDER (EU) and Junta Castilla-León (COVID19 grant COV20EDU/00187). Fundación Solórzano FS/38-2017.The Proteomics Unit belongs to ProteoRed, PRB3-ISCIII, supported by grant PT17/0019/0023, of the PE I + D + I 2017-2020, funded by ISCIII and FEDER. AL-V is supported by VIII Centenario-USAL PhD Program. PJ-V is supported by JCYL PhD Program and scholarship JCYL-EDU/601/2020. PD and EB are supported by a JCYL-EDU/346/2013 Ph.D. scholarship. The authors thank the Cell Sorting Service (NUCLEUS, University of Salamanca) for technical assistance.

## Conflict of Interest

The authors declare that the research was conducted in the absence of any commercial or financial relationships that could be construed as a potential conflict of interest.

## Supplementary Material

Supplementary Table 1 Qualitative results. The lists of identified proteins are shown for each B-cell subpopulation (naive B-cells, N (sheet 1); centroblasts, CB (sheet 2); centrocytes, CC (sheet 3); memory B-cells, M (sheet 4); plasma cells, PC (sheet 5)) including the protein identifier (Uniprot ID), complete name of the protein (description), abundance values for each B-cell subpopulation and replicate (1-5), and mean value. The sheet 6 contains the lists of proteins in common among B-cell subpopulations and the unique proteins per group resulting from a Venn analysis.

Supplementary Table 2 Unique proteins identified per B-cell subpopulation. Lists of proteins (gene name) exclusively identified in the indicated B-cell subpopulation are shown.

Supplementary Table 3 Quantitative analysis. The lists of proteins of the quantitative analysis are depicted (sheet 1) together with the 1-to-1 comparisons (sheets 2-7). The UniProt ID (accession), the number of detected peptides per protein (peptide count), the number of unique peptides per protein (unique peptides), the confidence score, the ANOVA p-value (ANOVA (p)), the max fold change, the B-cell population with the highest mean for each protein (highest mean condition), the B-cell population with the lowest mean for each protein (lowest mean condition), the complete name of the protein (description), and the normalized abundance for each replicate within each B-cell population are indicated. The significant proteins (ANOVA p-value < 0.05) detected after pair-wise comparisons of B-cell subpopulations. 1 means “significant differences”, 0 means “no significant differences” (sheet 8).

Supplementary Table 4 Quantitative protein results after comparing B-cell subpopulations with each other. The total number of significantly expressed proteins within each comparison is depicted as well as the number and percentage of proteins highly expressed in one subpopulation compared to the other one.

Supplementary Table 5 Functional enrichment analysis (FEA) of the proteins identified in the 5 B-cell subpopulations. The file contains 5 Tables with the annotated clusters (p-value<0.05): (I) Table “350 in common” shows the 56 annotated clusters identified for the proteins in common across the naïve (N), CB, CC, memory (M), and PC subsets. (II) Table “1547_N_CB_CC_M” includes the 129 annotated clusters identified for the proteins in common across N, CB, CC, and M subsets. (III) Table “419_CB_CC_M” displays the 63 annotated clusters for proteins in common in CB, CC, and M subsets. (IV) Table “77_CC_M” shows the two annotated clusters for proteins in common between CC and M subpopulations. (V) Table “25_PC” depicts the two annotated clusters identified for the 25 proteins exclusively detected in the PC subset. (VI) Lists of proteins identified within each B-cell subpopulation across the pathways represented in **Supplementary Figure S8**.

Supplementary Table 6 Significantly enriched modules and pathways related to the 5 B-cell subpopulations. The file contains 2 Tables: (I) Table “Modules” shows the different modules per B-cell subpopulation and the corresponding p-value, q-value and adjusted p-value, as well as the GeneName and GeneSymbol of the genes included in the module. (II) Table “Pathways” shows the same information as stated above for the pathways.

Supplementary Table 7 Data set missing proteins detected by MS/MS. Information on missing proteins detected after proteomic analyses in progress of B-lymphocyte differentiation during antigen-dependent phase.

Supplementary Table 8 Transcriptomic data. The file contains: (I) the results of the expression profiling of B-cell subsets sorted from human tonsils on Affymetrix Human Exon 1.0 ST arrays (raw data deposited at NCBI’s Gene Expression Omnibus database, GSE69033) after calculating the mean expression values (6 biological replicates) for each B-cell subset (naive B-cells, centroblasts, centrocytes, memory B-cells and plasma cells). The presence/absence of each gene displayed on the array in the proteomic analysis is also indicated. (II) The significant genes (ANOVA p-value < 0.05) detected after pair-wise comparisons of B-cell subpopulations. 1 means “significant differences”, 0 means “no significant differences”. (III) Lists of proteins, genes and their combination which have shown significant differences in the ANOVA test (p-value<0.05).

Supplementary Table 9 Proteomics/transcriptomics ratios. The correlation between both strategies was calculated according to the mean values for each gene/protein within each B-cell subpopulation. (I) Proteomics mean values for the 606 proteins having a transcriptomic counterpart in the expression microarray. (II) Proteomics/transcriptomics ratios for the 569 genes/proteins presenting values in both datasets.

Supplementary Table 10 Data set Proteomic clusters. Protein clusters were built by applying a hierarchical self-organizing map (SOM) algorithm and including Gene Ontology information. Each protein value was calculated as the mean protein expression in each B-cell population. Graphs showing these data are represented in **Supplementary Figure S11**.

Supplementary Figure 1 Proteomic workflow performed on B-cells. Panel **(A)** shows the B-cell ontogeny from the hematopoietic stem cell (HSC) to the long-lived B-cell populations. Panel **(B)** depicts the procedure followed to process the proteins from naive B-cells (N), centroblasts (CB), centrocytes (CC), memory B-cells (M) and plasma cells (PC) for further mass spectrometric (MS) analysis after in-solution and in-gel protein digestions.

Supplementary Figure 2 Gating strategy used for the classification of tonsilar B-cell populations. In a first step, total B-cells were subsequently gated as FSClow/SSClow, CD19+/CD20+/CD45+ and CD3- (pink dots) **(A)**. In a second step **(B)**, these gated B-cells were dissected into 5 subpopulations based on their staining profile for CD10, CD27, CD38, and CD184. CC: Centrocytes. CB: Centroblasts. MBC, Memory B-cells. PC, Plasma cells.

Supplementary Figure 3 Binary heatmap based on the presence/absence of the proteins identified in the B-cell subpopulations. The blue color represents the absence of a protein whereas the red color means presence.

Supplementary Figure 4 Principal component analysis (PCA) graphics of B-cell subpopulation comparisons after quantitative analysis of the identified proteins. The quantitative proteomics data used for this PCA is collected in **Supplementary Table S3**.

Supplementary Figure 5 Circular graphical representation of differentially expressed proteins **(A)**, genes **(B)** and the corresponding proteomics/transcriptomics ratios **(C)**. Each graphic has four layers. From the outer layer to the inner one, data for memory B-cells (orange), centrocytes (purple), centroblasts (blue) and naive (green) B-cells are represented. Each layer depicts the quantitative values for each protein within each chromosome. Remarkable proteins within the dataset are indicated in the graphic. Since it is difficult to read such protein names in the graphics, they are also mentioned here (in clockwise direction, same proteins/genes for panels **A–C**): THEMIS2, CD53, RGS13, TRAF3IP3 (chromosome 1); REL, XPO1, SF3B1 (chromosome 2); NFKB1 (chromosome 4); MZB1, CD74 (chromosome 5); HLA-B(x6), NFKBIE (chromosome 6); EZH2 (chromosome 7); LYN (chromosome 8); VIM, GDI1 (chromosome 10); CD44, MS4A1, CASP1 (chromosome 11); GAPDH (chromosome 12); TCL1A, HSP90AA1 (chromosome 14); GINS3, PSMD7, IRF8 (chromosome 16); STAT3 (chromosome 17); VAV1, DNMT1, VASP (chromosome 19); BID, MYH9 (chromosome 22).

Supplementary Figure 6 Diagram representation of stacked deviations of proteins identified and quantitatively measured in the B-cell subpopulations (naive B-cells, centroblasts, centrocytes, memory B-cells). The number of proteins that were differentially expressed, according to an outlier statistic, were calculated and represented in intervals of Z-distance from the mean.

Supplementary Figure 7 Attribute plots of pair-wise comparisons of **(A)** proteins (data connected to **Supplementary Tables S3** and **S4**) and **(B)** genes (data connected to **Supplementary Table S8**) differentially expressed (ANOVA p-value < 0.05) across the B-cell subpopulations. Set size refers to the total number of proteins **(A)** or genes **(B)** present in each pair of populations (e.g. almost 600 proteins in common for CC and M). Intersection size depicts the number of proteins or genes in common for the paired populations only (orange, brown, pink, grey, black and yellow for CC-M, CB-M, N-CC, N-CB, CB-CC and N-M, respectively) or their combinations (bars in dark grey, combinations of paired-populations indicated by black dots connected by black lines in the bottom part of the graph). The bigger the intersection size, the more proteins/genes detected in those paired-populations or combinations of paired-populations. N, naive B-cells; M, memory B-cells; CB, centroblasts; CC, centrocytes.

Supplementary Figure 8 Graphical visualization of the protein involvement in signaling pathways. Bars length represents the total number of proteins contained in the given pathways: in green (proteins of naive B-cells), blue (proteins of centroblasts), light purple (proteins of centrocytes), light orange (proteins of memory B-cells), red (proteins of plasma cells), and brown (proteins in common). The related lists of proteins are collected in **Supplementary Table S5**.

Supplementary Figure 9 Significantly enriched modules and pathways. Panel **(A)** shows the modules detected as significantly enriched across the 5 B-cell subpopulations. Panel **(B)** displays the significantly enriched pathways. The size of the dots correlates with the number of genes identified in the corresponding B-cell population belonging to the referenced module. The adjusted p-value is color-coded from 0.01 and 0.004 (red) to 0.04 and 0.016 (blue) for modules (panel **A**) and pathways (panel **B**), respectively. s.p., signaling pathway.

Supplementary Figure 10 Mean protein expression pattern of missing proteins detected for MS/MS in B-cell populations. The mean intensity of the 5 replicates for each B-cell population was represented by family groups. N, naive B-cells; CB, centroblasts; CC, centrocytes; M, memory B-cells.

Supplementary Figure 11 Proteomic mean expression. The figure shows clusters of proteins built by applying a hierarchical self-organizing map (SOM) algorithm and including Gene Ontology information (molecular function and/or subcellular location). Each protein value in each cluster corresponds to the mean protein expression level (information collected in **Supplementary Table S10**, original data in **Supplementary Table S3**) across the five biological replicates analyzed per B-cell subpopulation. The figure shows how these proteins are differently expressed in naïve (N), centroblast (CB), centrocyte (CC) and memory B-cell (M) populations.

Supplementary Figure 12 Comparative analysis of specific proteins investigated by flow cytometry (FCM) and mass spectrometry (MS/MS) approaches. The intensity values obtained with each strategy are represented in each graphic for each evaluated protein. The gray line represents the FCM data, whereas the orange one depicts the MS/MS results. N, naive B-cells; CB, centroblast; CC, centrocyte; M, memory B-cell.

Supplementary File 1 R Script for the processing and analysis of proteomic MS/MS data and transcriptomic microarray results and their ulterior multi-omics integration for the study of the dynamics of B-cell populations.

## Abbreviations

Ag, Antigen; BCR, B-cell receptor; CB, Centroblasts; CC, Centrocytes; FDR, False discovery rate; GC, Germinal centers; Igs, Immunoglobulins; MCL, Mantle cell lymphoma; M, Memory B-cell; N, Naive B-cell; PC, Plasma cells; PCA, Principal component analysis; PB, Peripheral blood; RT, Room temperature; SLT, Secondary lymphoid tissues; SHM, Through somatic hypermutation.
