# Supplementary material for: Dynamic Intracellular Metabolic Cell Signaling Profiles During Ag-Dependent B-Cell Differentiation
Source: Front Immunol. 2021 Mar 30;12:637832. doi: 10.3389/fimmu.2021.637832 (PMC8043114; doi:10.3389/fimmu.2021.637832)
Supplement: Supplementary Table 2 — Unique proteins identified per B-cell subpopulation. Lists of proteins (gene name) exclusively identified in the indicated B-cell subpopulation are shown. [file Table_2.docx]

| B-cell subpopulation | Gene name |  |
| --- | --- | --- |
| Naive B cells (n=7) | C9orf64; HBQ1; MRPL21; NDUFA3; PCBD2;PURB; RNASEH2C | |
| Centroblasts (n=24) | ARMT1; ASH2L; BRD2; CARMIL2; CLPTM1; DENND4B; DNAJC13; ECM29; FADD; GINS1; GMEB2; GOSR2; GTF3C3; HIGD1A; JUP; LRWD1; NAF1; RAP1GDS1; RPP25L; SYNE3; TMA7; UL1; VAV1; VAV2 | |
| Centrocytes (n=50) | ACSF2; AMPH; ASB2; ALKBH5; AVL9BCL7A; C9orf114; C17orf75; CCDC126; CCT6B; CNOT10; CRK; CWC27; DGKA; FAF1; GOLT1B; GTPBP3; HIST2H2AB; KAT7; KIAA0196; LIN7C; LINC00523; MED31; MRI1; MRPL9; MRPS9; NCF2; NDUFB11; NIF3L1; NPC2; NR3C1; NUGGC; PAWR; PEG10; PIP; PRKRA; RGS13; RHEB; RNF181; RPL3L; SORD; STAMBPL1; STX8; SYAP1; TSEN15; TSR2; UBXN6; VKORC1L1; VWA9; WDR75 | |
| Memory B cells (n=48) | AAAS; AGPAT1; ANAPC1; BAG5; BCL2L13; CCDC53; CNFN; COL11A1; EBF1; ELAC2; FAM210A; FOXK1; FXR2; GIGYF2; GNAS; GNPDA1; GTF3C1; ICAM3; IFIT5; INTS3; IRF9; ITGB1; KNTC1; KYNU; LGMN; LRBA; MAPKAP1; MAPK9; MRPL55; MRPS35; NEDD8; NMI; NT5C2; NT5C3A; NUDT16; PRAF2; PSAP; PTDSS1; RASSF5; ROCK1; RPE; SAMM50; SPTLC1; STXBP2; THEMIS2; THG1L; UNC93B1; YARS2 | |
| Plasma cells (n=25) | ABI1; ARFGAP3; CLTB; CNBP; COX6B1; COX7C; DBI; FAM133A; GTF2A1; HDLBP; HIST1H2BJ; HN1; HN1L; IGHD; IGKV3-11; IGKV3-15; IGLC3; IRF4; JCHAIN; KIF5B; LZTFL1; SEC61G; SH3GLB2; SNRPC; UBE2D1 | |

**Supplemental Table S2.** **Unique proteins identified per B-cell subpopulation.** Lists of proteins (gene name) exclusively identified in the indicated B-cell subpopulation are shown.
