# Supplementary material for: Dynamic Intracellular Metabolic Cell Signaling Profiles During Ag-Dependent B-Cell Differentiation
Source: Front Immunol. 2021 Mar 30;12:637832. doi: 10.3389/fimmu.2021.637832 (PMC8043114; doi:10.3389/fimmu.2021.637832)
Supplement: Supplementary Table 4 — Quantitative protein results after comparing B-cell subpopulations with each other. The total number of significantly expressed proteins within each comparison is depicted as well as the number and percentage of proteins highly expressed in one subpopulation compared to the other one. [file Table_4.docx]

| B-cell subpopulation A | B-cell subpopulation B | No. of total significantly expressed proteins^a^ | No. of highly expressed proteins in subpopulation A^b^ | No. of highly expressed proteins in subpopulation B^b^ |
| --- | --- | --- | --- | --- |
| N | **CB** | 511 | 18 (4%) | 493 (96%) |
| N | **CC** | 609 | 16 (3%) | 593 (97%) |
| N | **M** | 299 | 29 (10%) | 270 (90%) |
| CB | **CC** | 171 | 103 (60%) | 68 (40%) |
| CB | **M** | 456 | 359 (79%) | 97 (21%) |
| CC | **M** | 615 | 469 (76%) | 146 (24%) |

^a^ p-value < 0.05

^b^ In brackets it is shown the percentage of highly expressed proteins with respect to the total of significantly expressed proteins within each comparison.

N, naive B cells; CB, centroblasts; CC, centrocytes; M, memory B cells.
