# Supplementary figures and images for: Dynamic Intracellular Metabolic Cell Signaling Profiles During Ag-Dependent B-Cell Differentiation
Source: Front Immunol. 2021 Mar 30;12:637832. doi: 10.3389/fimmu.2021.637832 (PMC8043114; doi:10.3389/fimmu.2021.637832)

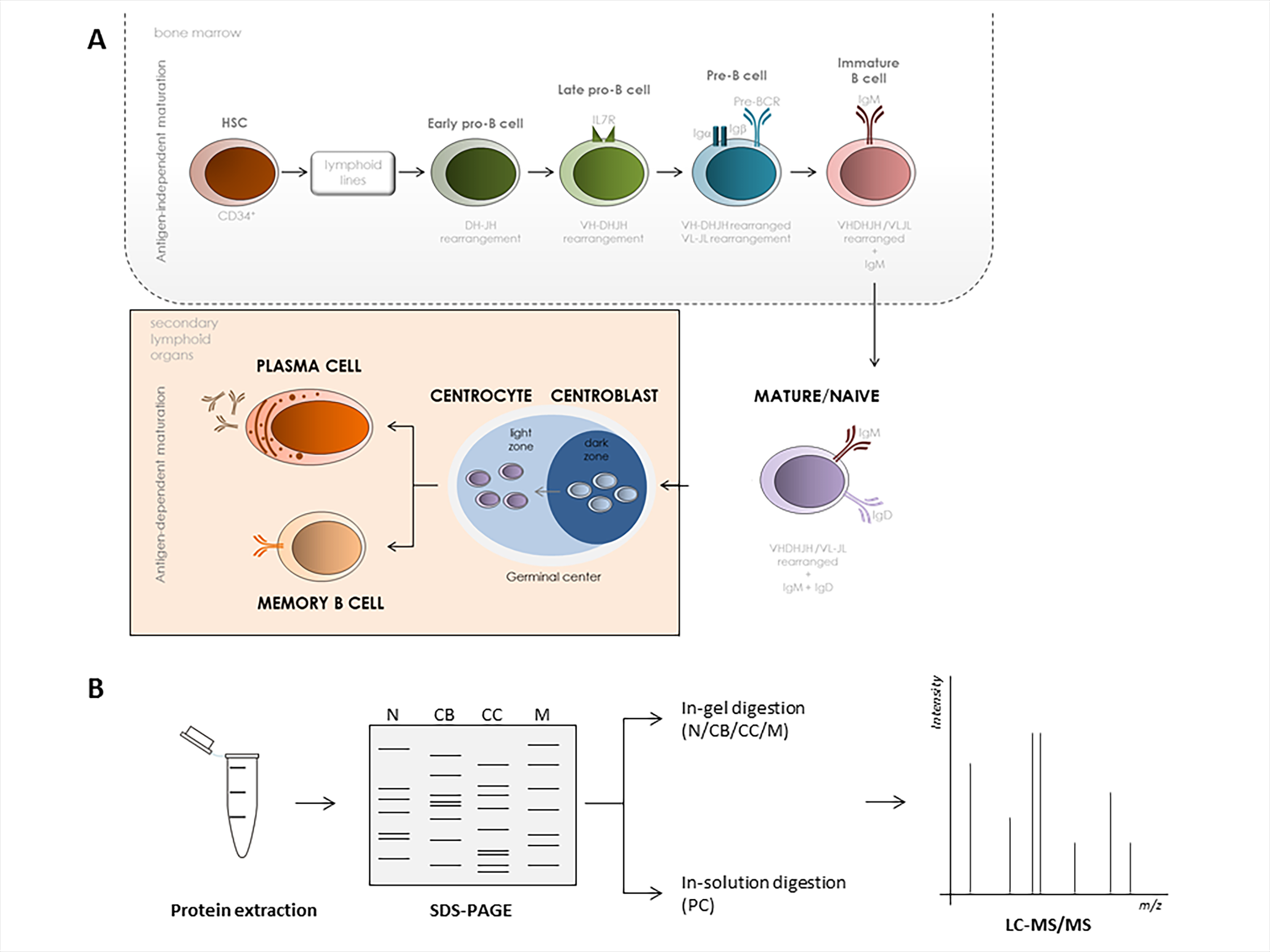

Supplement: Supplementary Figure 1 — Proteomic workflow performed on B-cells. Panel (A) shows the B-cell ontogeny from the hematopoietic stem cell (HSC) to the long-lived B-cell populations. Panel (B) depicts the procedure followed to process the proteins from naive B-cells (N), centroblasts (CB), centrocytes (CC), memory B-cells (M) and plasma cells (PC) for further mass spectrometric (MS) analysis after in-solution and in-gel protein digestions. [file Image_1.tif]

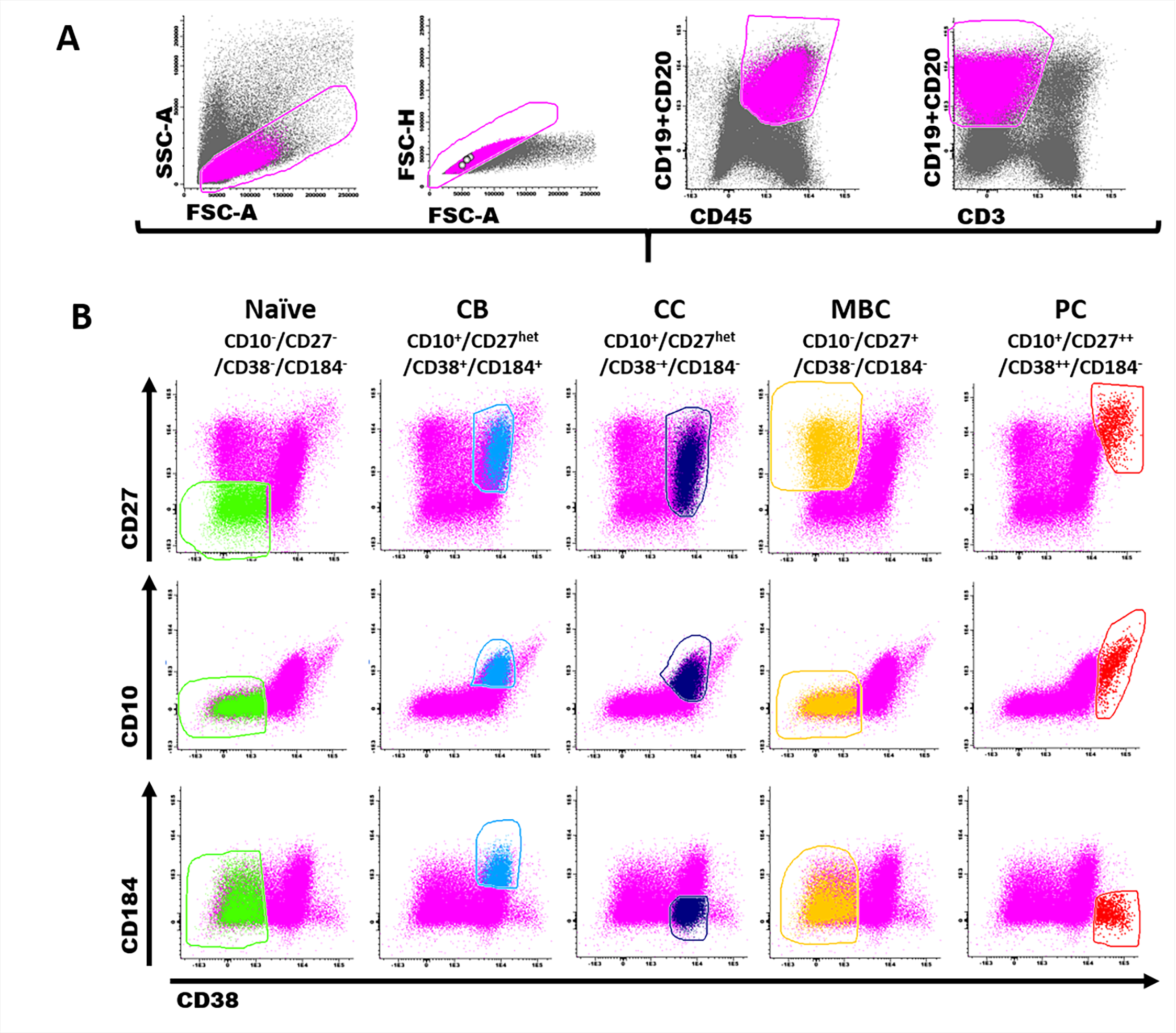

Supplement: Supplementary Figure 2 — Gating strategy used for the classification of tonsilar B-cell populations. In a first step, total B-cells were subsequently gated as FSClow/SSClow, CD19+/CD20+/CD45+ and CD3- (pink dots) (A). In a second step (B), these gated B-cells were dissected into 5 subpopulations based on their staining profile for CD10, CD27, CD38, and CD184. CC: Centrocytes. CB: Centroblasts. MBC, Memory B-cells. PC, Plasma cells. [file Image_2.tif]

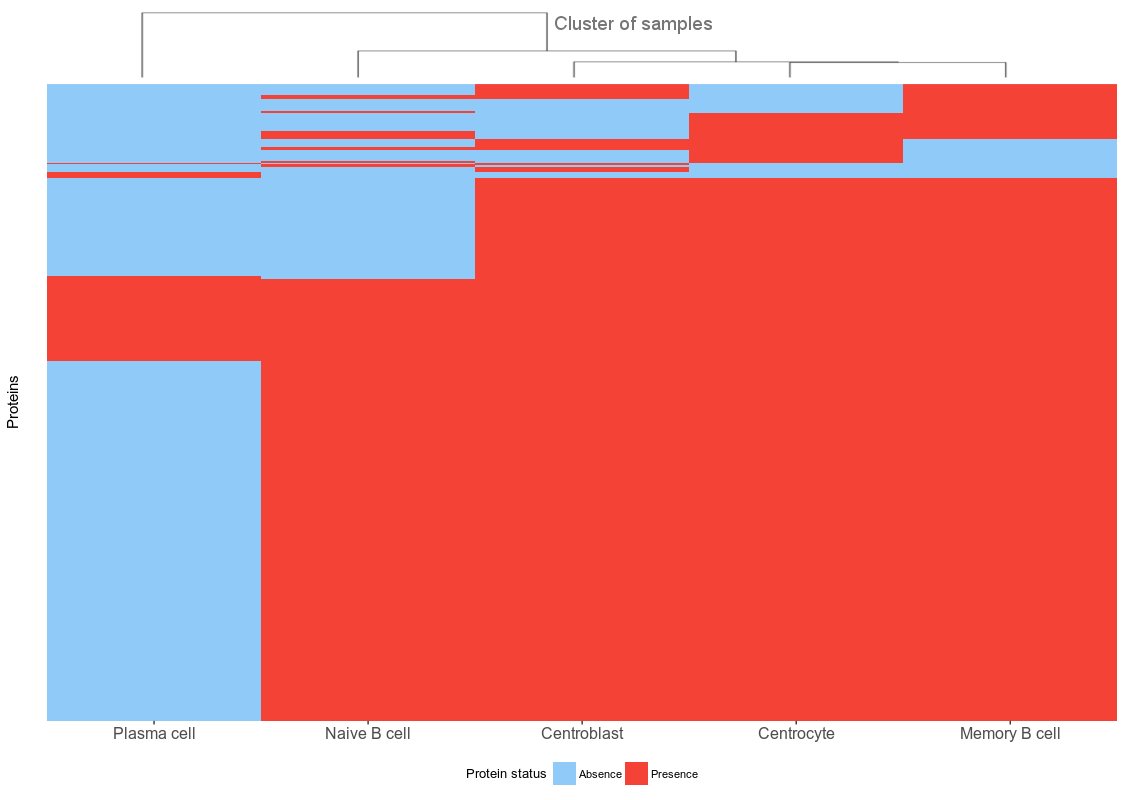

Supplement: Supplementary Figure 3 — Binary heatmap based on the presence/absence of the proteins identified in the B-cell subpopulations. The blue color represents the absence of a protein whereas the red color means presence. [file Image_3.tiff]

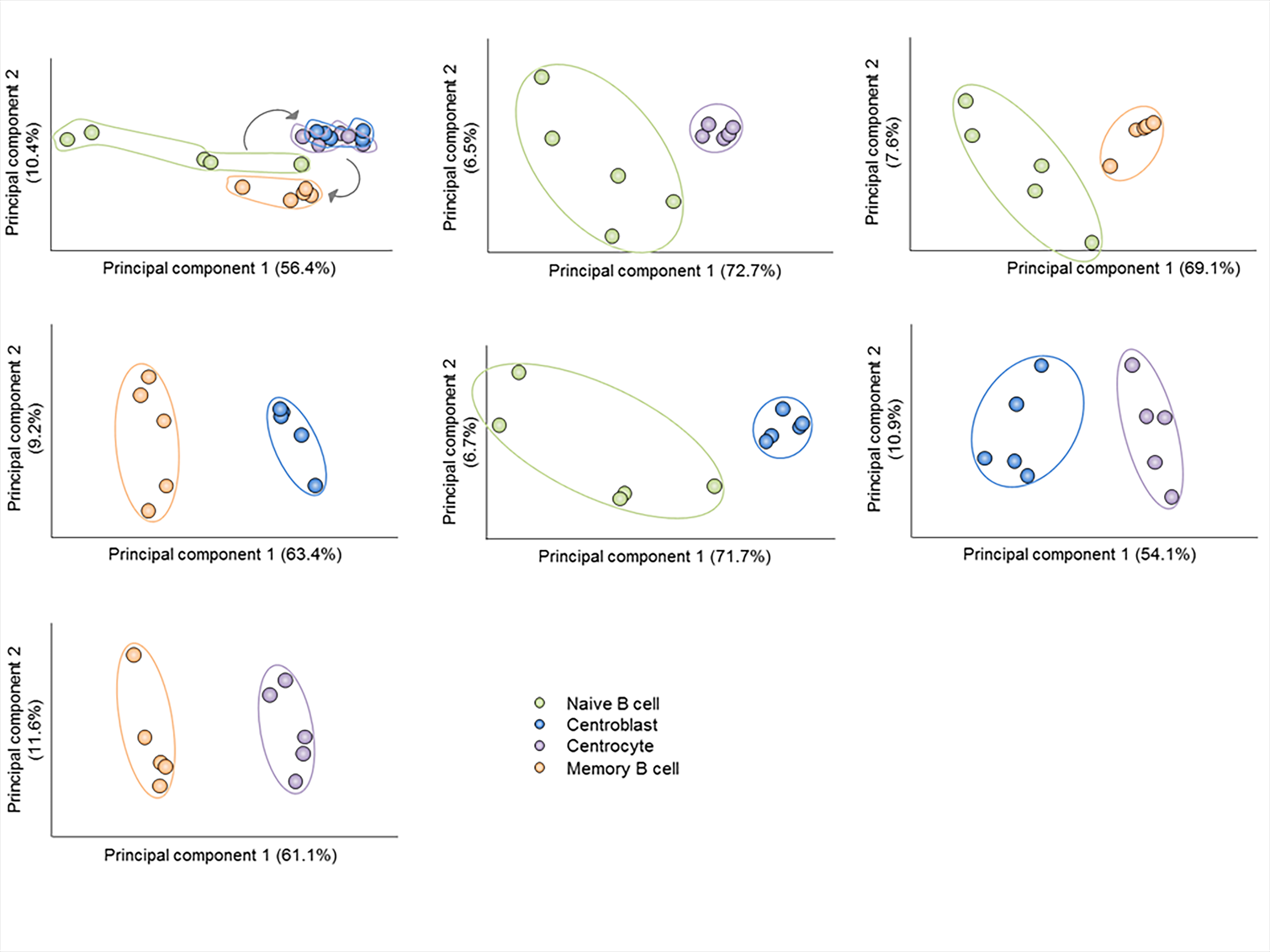

Supplement: Supplementary Figure 4 — Principal component analysis (PCA) graphics of B-cell subpopulation comparisons after quantitative analysis of the identified proteins. The quantitative proteomics data used for this PCA is collected in Supplementary Table S3 . [file Image_4.tif]

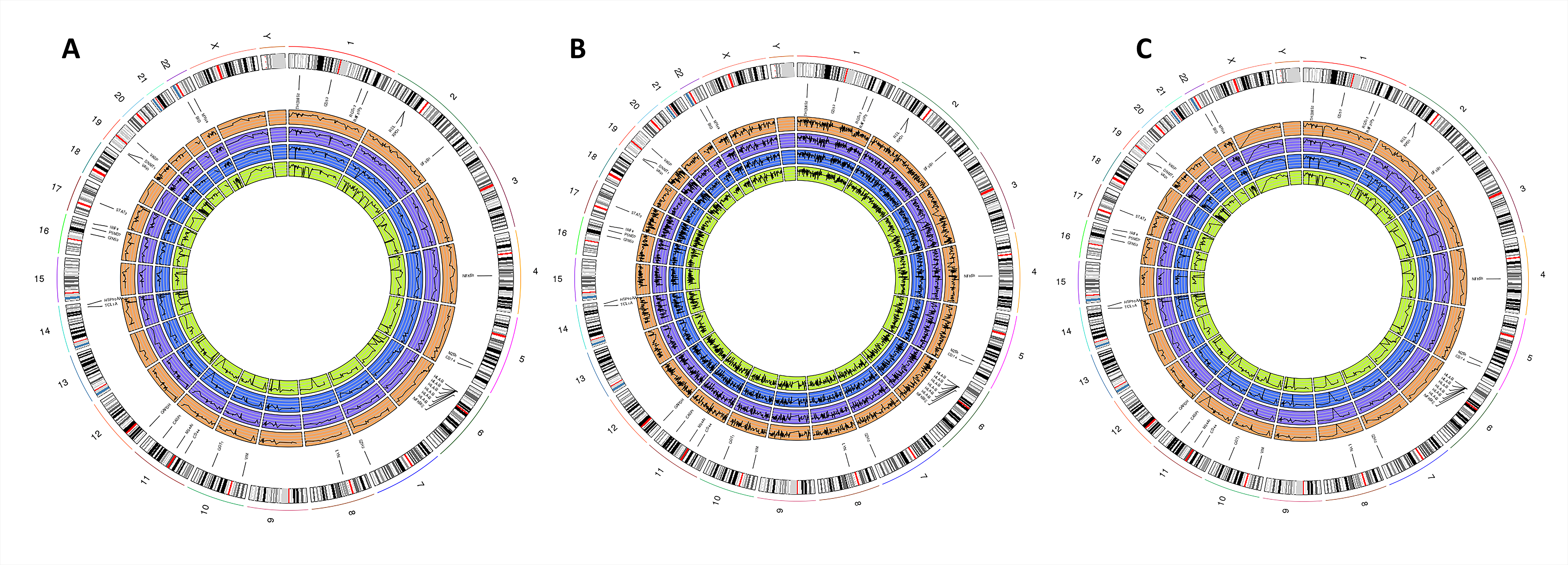

Supplement: Supplementary Figure 5 — Circular graphical representation of differentially expressed proteins (A), genes (B) and the corresponding proteomics/transcriptomics ratios (C). Each graphic has four layers. From the outer layer to the inner one, data for memory B-cells (orange), centrocytes (purple), centroblasts (blue) and naive (green) B-cells are represented. Each layer depicts the quantitative values for each protein within each chromosome. Remarkable proteins within the dataset are indicated in the graphic. Since it is difficult to read such protein names in the graphics, they are also mentioned here (in clockwise direction, same proteins/genes for panels A–C): THEMIS2, CD53, RGS13, TRAF3IP3 (chromosome 1); REL, XPO1, SF3B1 (chromosome 2); NFKB1 (chromosome 4); MZB1, CD74 (chromosome 5); HLA-B(x6), NFKBIE (chromosome 6); EZH2 (chromosome 7); LYN (chromosome 8); VIM, GDI1 (chromosome 10); CD44, MS4A1, CASP1 (chromosome 11); GAPDH (chromosome 12); TCL1A, HSP90AA1 (chromosome 14); GINS3, PSMD7, IRF8 (chromosome 16); STAT3 (chromosome 17); VAV1, DNMT1, VASP (chromosome 19); BID, MYH9 (chromosome 22). [file Image_5.tif]

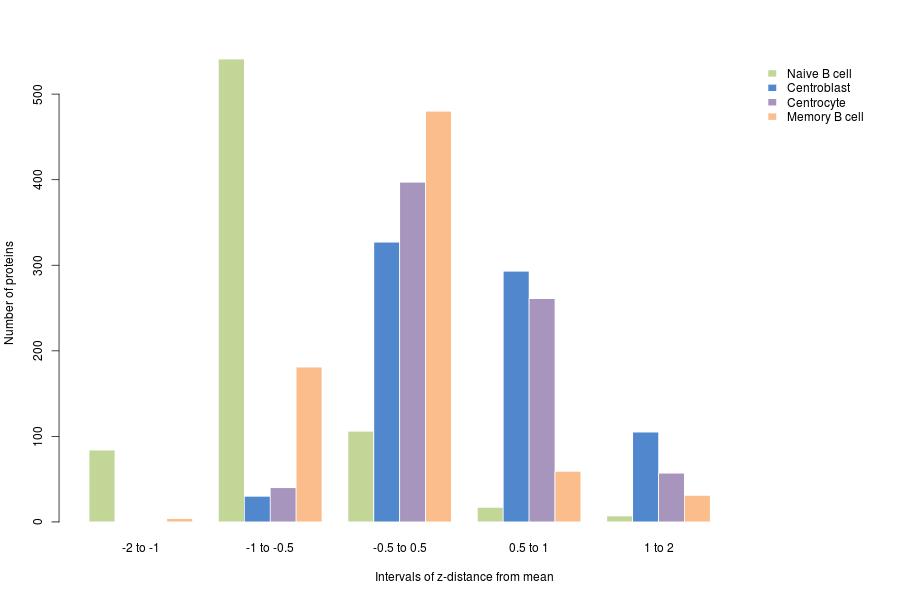

Supplement: Supplementary Figure 6 — Diagram representation of stacked deviations of proteins identified and quantitatively measured in the B-cell subpopulations (naive B-cells, centroblasts, centrocytes, memory B-cells). The number of proteins that were differentially expressed, according to an outlier statistic, were calculated and represented in intervals of Z-distance from the mean. [file Image_6.tiff]

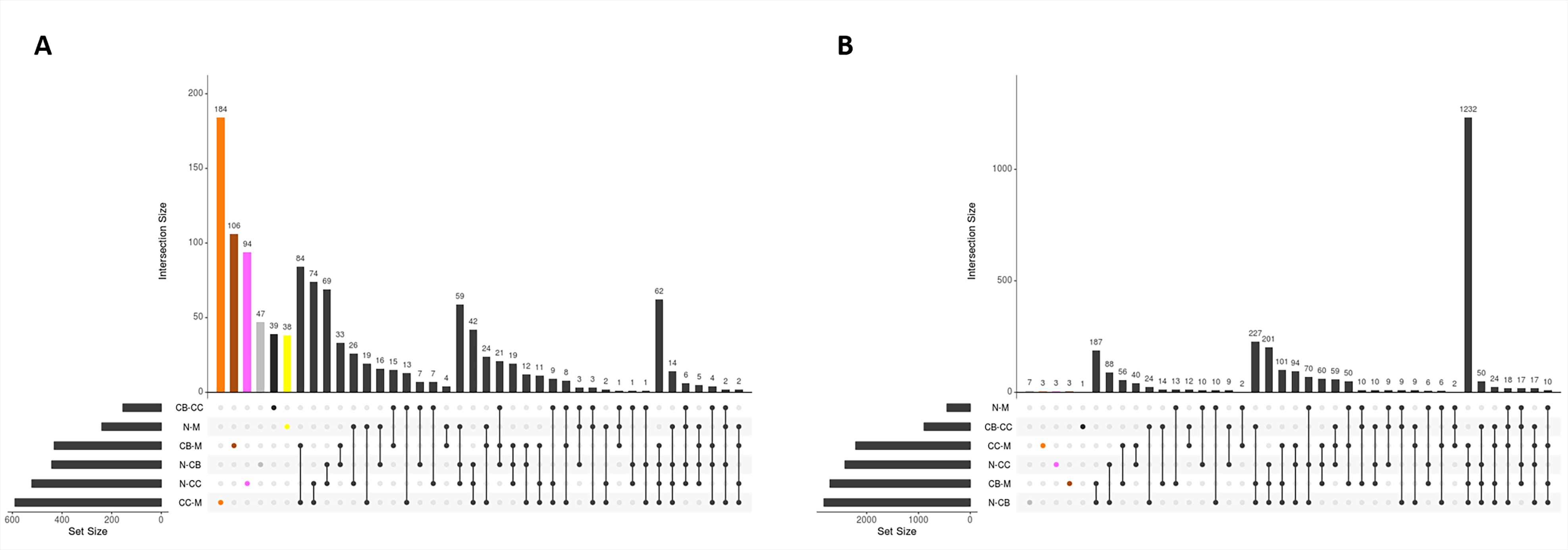

Supplement: Supplementary Figure 7 — Attribute plots of pair-wise comparisons of (A) proteins (data connected to Supplementary Tables S3 and S4 ) and (B) genes (data connected to Supplementary Table S8 ) differentially expressed (ANOVA p-value < 0.05) across the B-cell subpopulations. Set size refers to the total number of proteins (A) or genes (B) present in each pair of populations (e.g. almost 600 proteins in common for CC and M). Intersection size depicts the number of proteins or genes in common for the paired populations only (orange, brown, pink, grey, black and yellow for CC-M, CB-M, N-CC, N-CB, CB-CC and N-M, respectively) or their combinations (bars in dark grey, combinations of paired-populations indicated by black dots connected by black lines in the bottom part of the graph). The bigger the intersection size, the more proteins/genes detected in those paired-populations or combinations of paired-populations. N, naive B-cells; M, memory B-cells; CB, centroblasts; CC, centrocytes. [file Image_7.tif]

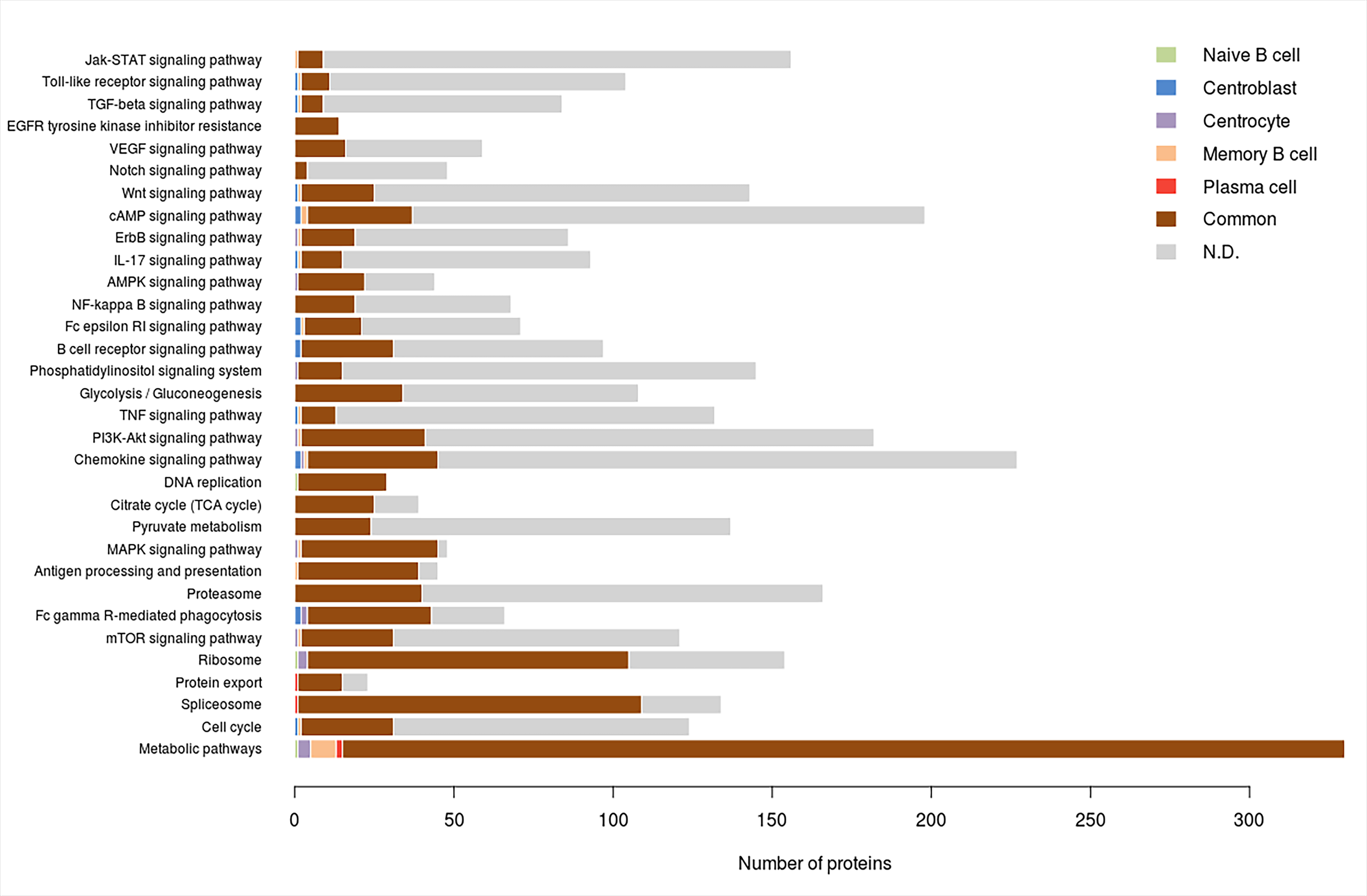

Supplement: Supplementary Figure 8 — Graphical visualization of the protein involvement in signaling pathways. Bars length represents the total number of proteins contained in the given pathways: in green (proteins of naive B-cells), blue (proteins of centroblasts), light purple (proteins of centrocytes), light orange (proteins of memory B-cells), red (proteins of plasma cells), and brown (proteins in common). The related lists of proteins are collected in Supplementary Table S5 . [file Image_8.tif]

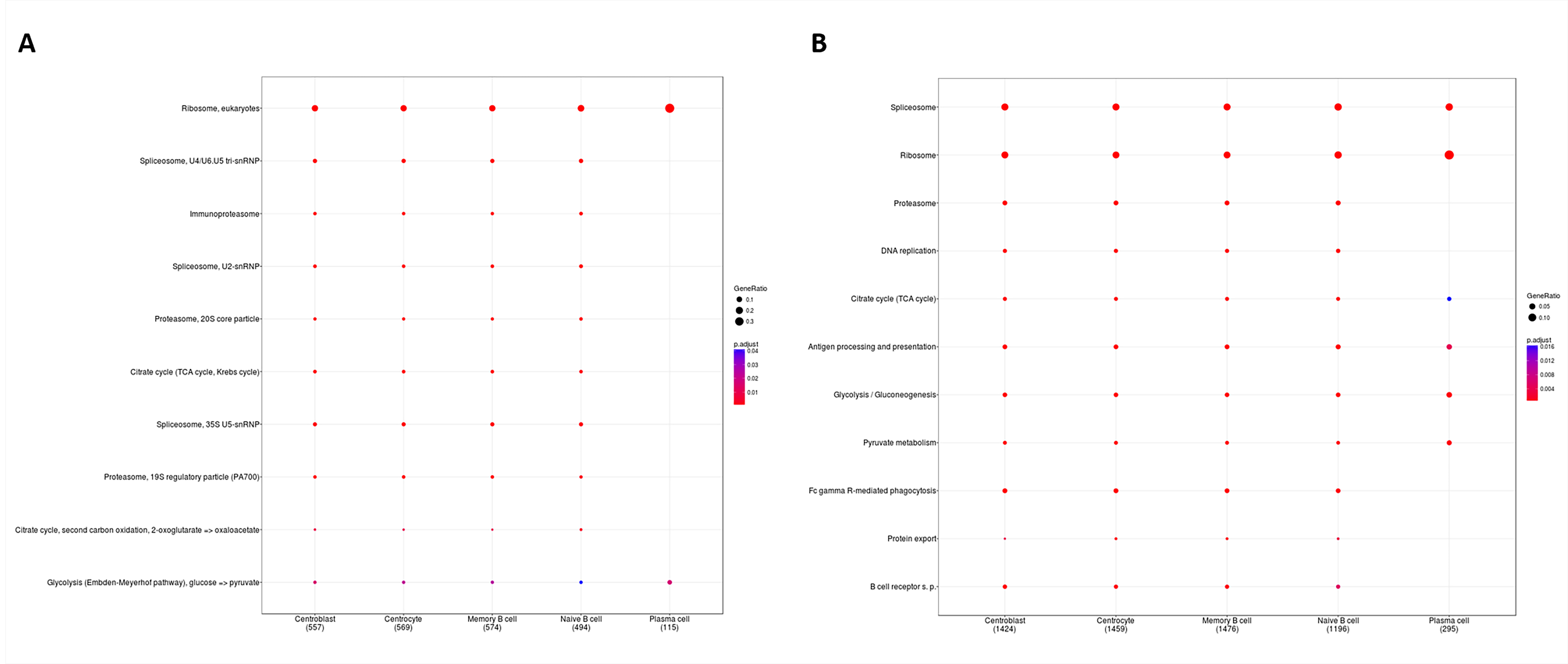

Supplement: Supplementary Figure 9 — Significantly enriched modules and pathways. Panel (A) shows the modules detected as significantly enriched across the 5 B-cell subpopulations. Panel (B) displays the significantly enriched pathways. The size of the dots correlates with the number of genes identified in the corresponding B-cell population belonging to the referenced module. The adjusted p-value is color-coded from 0.01 and 0.004 (red) to 0.04 and 0.016 (blue) for modules (panel A) and pathways (panel B), respectively. s.p., signaling pathway. [file Image_9.tif]

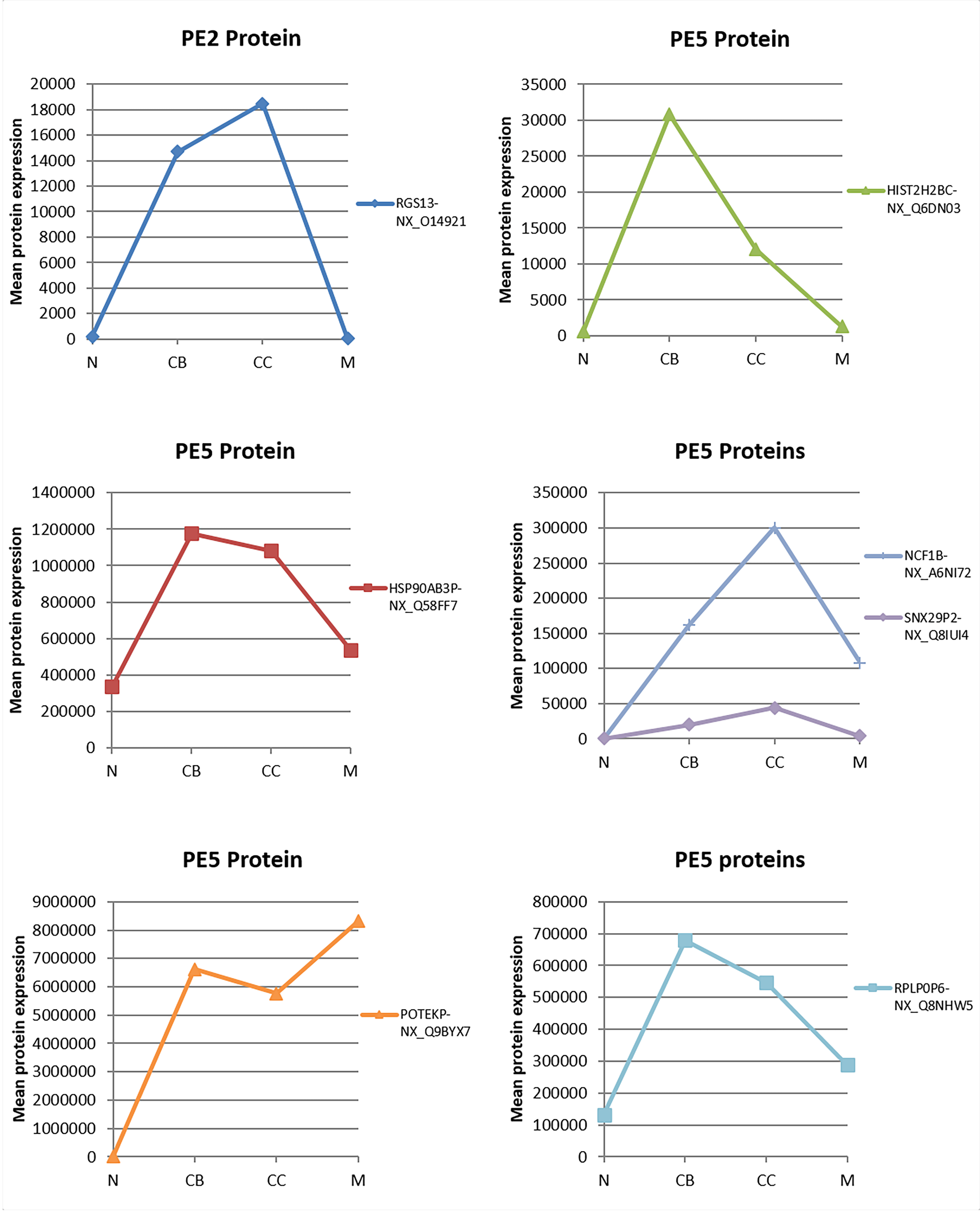

Supplement: Supplementary Figure 10 — Mean protein expression pattern of missing proteins detected for MS/MS in B-cell populations. The mean intensity of the 5 replicates for each B-cell population was represented by family groups. N, naive B-cells; CB, centroblasts; CC, centrocytes; M, memory B-cells. [file Image_10.tif]

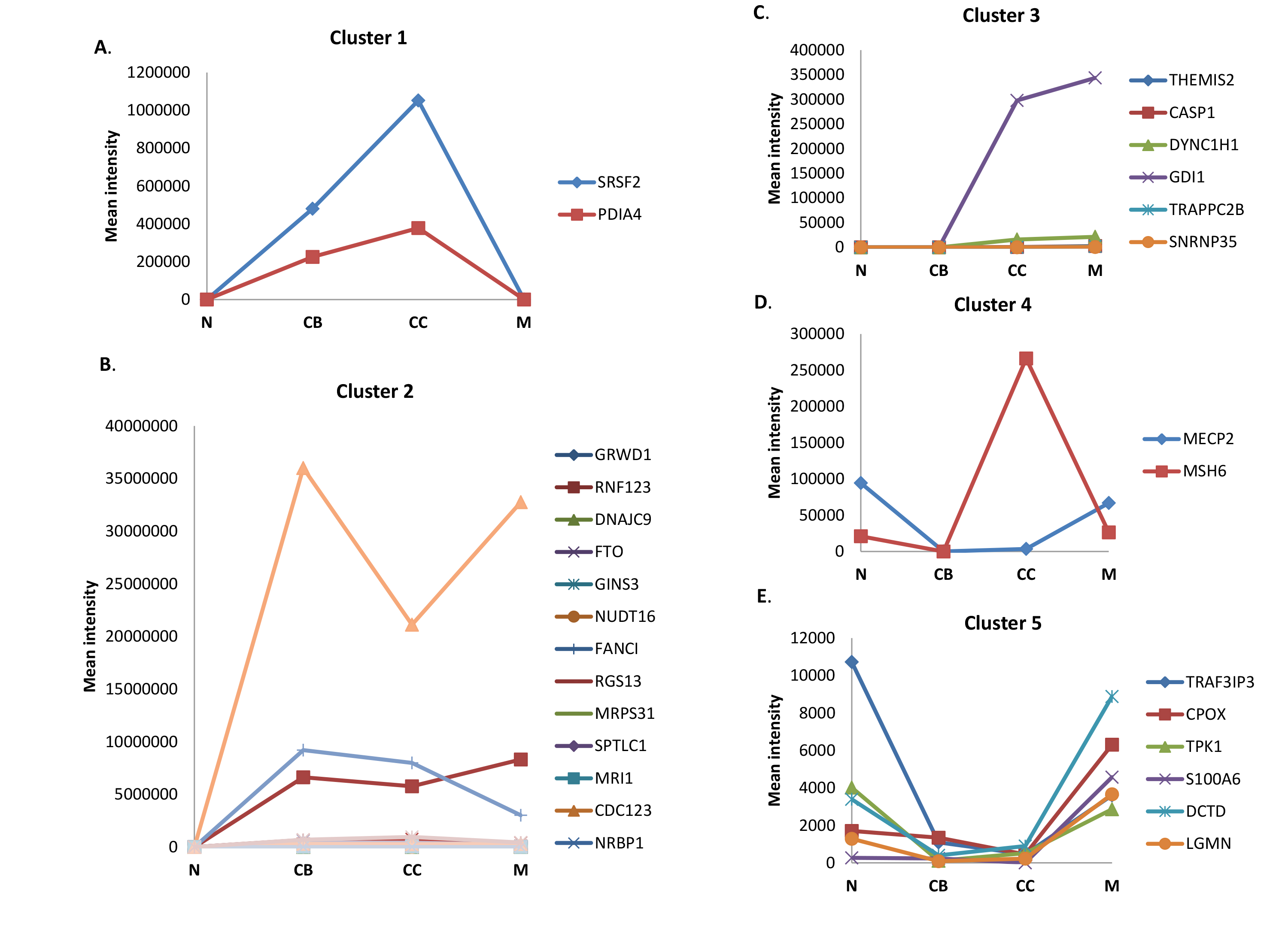

Supplement: Supplementary Figure 11 — Proteomic mean expression. The figure shows clusters of proteins built by applying a hierarchical self-organizing map (SOM) algorithm and including Gene Ontology information (molecular function and/or subcellular location). Each protein value in each cluster corresponds to the mean protein expression level (information collected in Supplementary Table S10 , original data in Supplementary Table S3 ) across the five biological replicates analyzed per B-cell subpopulation. The figure shows how these proteins are differently expressed in naïve (N), centroblast (CB), centrocyte (CC) and memory B-cell (M) populations. [file Image_11.tif]

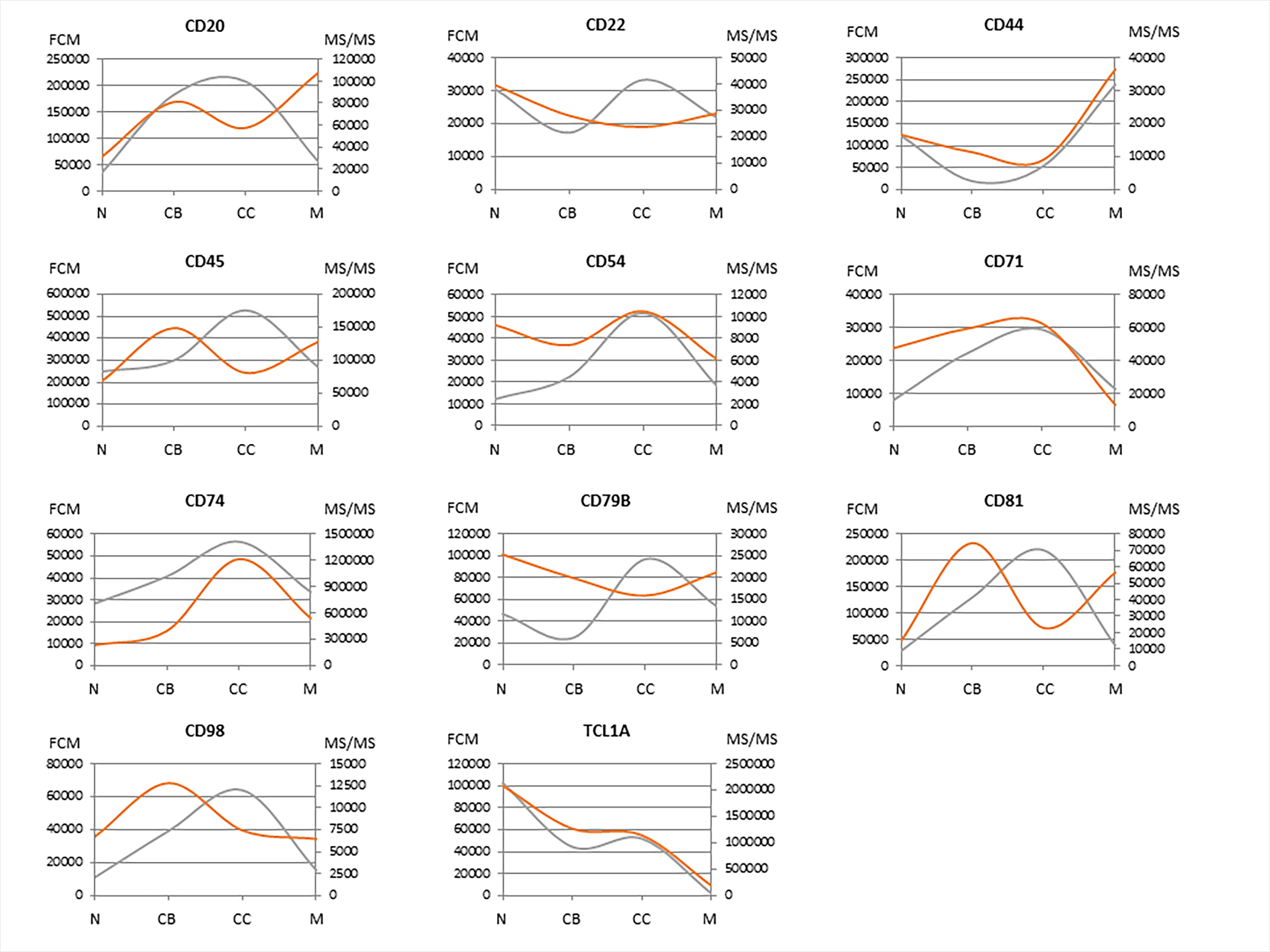

Supplement: Supplementary Figure 12 — Comparative analysis of specific proteins investigated by flow cytometry (FCM) and mass spectrometry (MS/MS) approaches. The intensity values obtained with each strategy are represented in each graphic for each evaluated protein. The gray line represents the FCM data, whereas the orange one depicts the MS/MS results. N, naive B-cells; CB, centroblast; CC, centrocyte; M, memory B-cell. [file Image_12.tif]
